# Hippocampus-retrosplenial cortex interaction is increased during phasic REM and contributes to memory consolidation

**DOI:** 10.1038/s41598-021-91659-5

**Published:** 2021-06-22

**Authors:** Daniel Gomes de Almeida-Filho, Bruna Del Vechio Koike, Francesca Billwiller, Kelly Soares Farias, Igor Rafael Praxedes de Sales, Pierre-Hervé Luppi, Sidarta Ribeiro, Claudio Marcos Queiroz

**Affiliations:** 1 Brain Institute, Federal University of Rio Grande do Norte, Natal, RN Brazil; 2 UMR 5292 CNRS/U1028 INSERM, Center of Research in Neuroscience of Lyon, SLEEP Team, Université Claude Bernard Lyon I, Faculté de Médecine RTH Laennec, Lyon, France; 3 Federal University of the São Francisco Valley, Petrolina, PE Brazil; 4 State University of Paraíba, Campina Grande, PB Brazil; 5 Present Address: Department of Neurobiology, Integrative Center for Learning and Memory, Brain Research Institute, University of California, Los Angeles, CA 90095 USA

**Keywords:** Learning and memory, Consolidation, Cortex, Fear conditioning, Hippocampus, Circadian rhythms and sleep, REM sleep

## Abstract

Hippocampal (HPC) theta oscillation during post-training rapid eye movement (REM) sleep supports spatial learning. Theta also modulates neuronal and oscillatory activity in the retrosplenial cortex (RSC) during REM sleep. To investigate the relevance of theta-driven interaction between these two regions to memory consolidation, we computed the Granger causality within theta range on electrophysiological data recorded in freely behaving rats during REM sleep, both before and after contextual fear conditioning. We found a training-induced modulation of causality between HPC and RSC that was correlated with memory retrieval 24 h later. Retrieval was proportional to the change in the relative influence RSC exerted upon HPC theta oscillation. Importantly, causality peaked during theta acceleration, in synchrony with phasic REM sleep. Altogether, these results support a role for phasic REM sleep in hippocampo-cortical memory consolidation and suggest that causality modulation between RSC and HPC during REM sleep plays a functional role in that phenomenon.

## Introduction

Neuronal networks comprising the hippocampus (HPC) and selected neocortical regions such as the retrosplenial cortex (RSC) have been considered essential for the encoding, consolidation, and gradual corticalization of spatial memory traces^1–4^. Although the mechanisms responsible for the spreading of memory traces from the HPC to the neocortex are not entirely clear^4^, it has been suggested that hippocampal oscillations during sleep could boost synaptic plasticity in extra-hippocampal networks so as to facilitate systems consolidation^5^.

Two distinct electrophysiological patterns observed in the HPC of rodents during sleep have been consistently related to memory processing: the sharp-wave ripple (SWR) complex^6^, and the sustained theta oscillation^7^. While the former is mainly observed during slow-wave sleep (SWS), the latter is the hallmark of rapid eye movement (REM, a.k.a. paradoxical) sleep in rodents^7^. Hippocampal neuronal patterns exhibited during the waking experience are replayed during SWR events^8^, and HPC-RSC projections coordinate RSC neuronal activity during hippocampal SWR^9^. Additionally, abolishing the SWR complex during SWS impairs spatial memory^10^. Similarly, blockage of the sustained theta oscillation during REM sleep impairs contextual memory expression^11^. While the synchronization of SWR with neocortical ripples and spindles during SWS has been largely implicated in spatial memory processing^12^, there is scarce direct evidence relating REM sleep theta oscillation to the phenomenon of memory corticalization^13^.

Interestingly, the sustained theta oscillation observed during REM sleep can briefly hasten. Named as phasic REM sleep (phREM)^14^, these events are characterized by transient acceleration of the theta rhythm lasting ~ 2 s in which hippocampal neuronal activity boosts^14^. Also, phREM events show increased power of high-frequency oscillations and enhanced theta- and gamma-band coherence between and within the hippocampal tri-synaptic pathway^14,15^. While phREM events have been hypothesized to be related to memory processing, and to constitute a window for information output from the HPC to cortical targets^14^, direct evidence relating phREM events and memory processing is still lacking.

We have recently reported that HPC theta oscillation during REM sleep modulates oscillatory and neuronal activity within the RSC^16^. Both regions are related to contextual fear memory processing^1^, and their cross-talk has been related to memory performance^2,3,17,18^. Although already hypothesized^5,16^, the link between HPC-RSC interplay during REM sleep with memory processing is yet to be demonstrated. Here, we used Granger causality (GC) to quantify the interaction between local field potential recordings from the HPC and the RSC during REM sleep immediately before and after contextual fear conditioning (CFC), an experience with high emotional content. The GC method is widely used to infer the strength and direction of the exchanged influence between time series in the time and frequency domains^19,20^. In our dataset, we observed changes in causality estimates within the spectral range of theta rhythm between HPC and RSC during REM sleep, which correlated with memory performance tested 24 h after training. Interestingly, phREM events coincided with augmented exchanged influence in the HPC-RSC network. These results point to a specific role of REM sleep theta oscillations in spatial memory processing, mainly conveyed by the HPC-RSC intercommunication during phREM events.

## Results

### CFC elicits fear behavior 24 h after training

**Figure 1:**
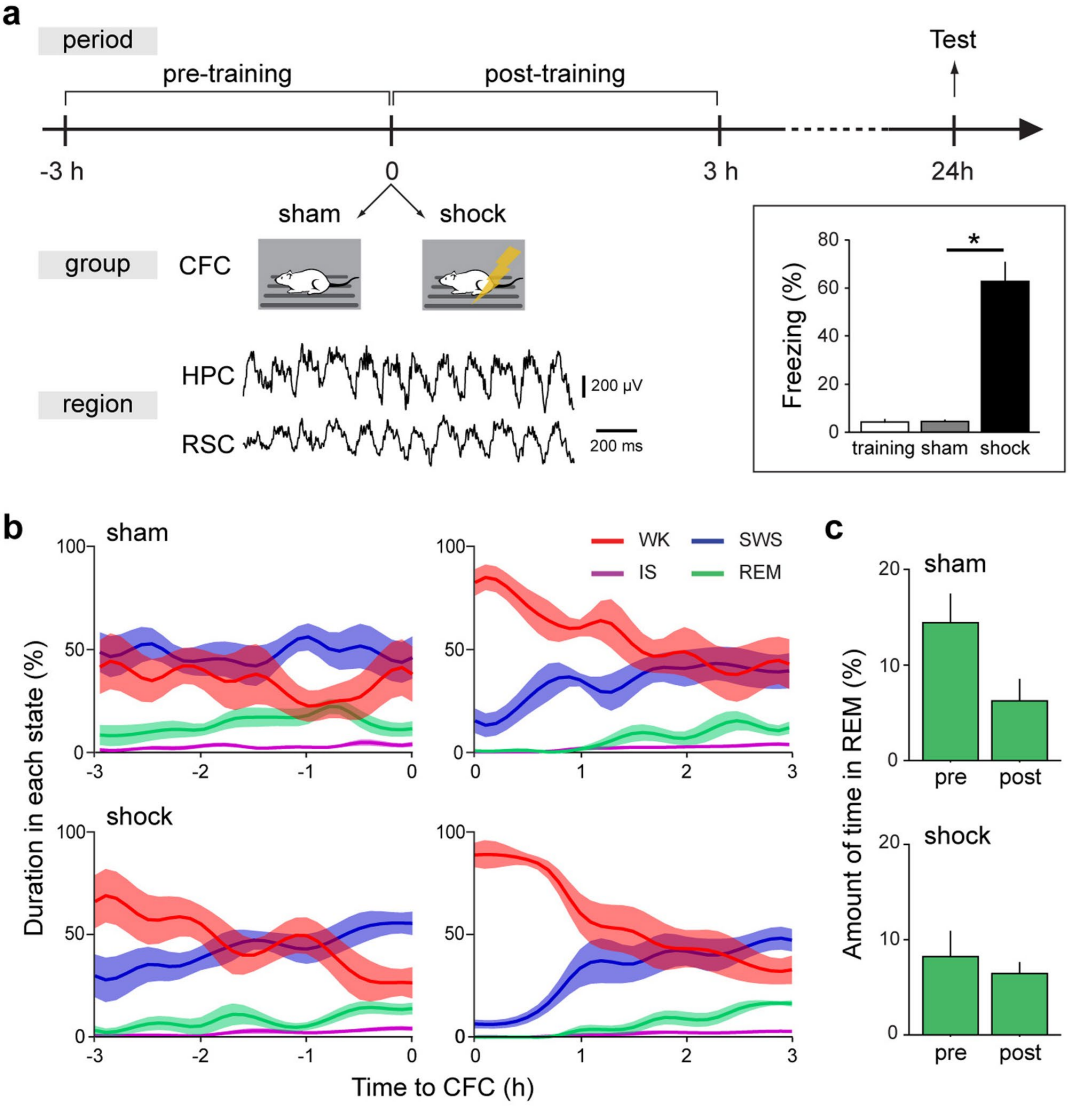
Experimental protocol and sleep architecture. (**a**) Experimental design and results. Animals (*N* = 13) were implanted with matrices of single wires into the hippocampus (HPC) and the retrosplenial cortex (RSC), and recorded 3 h before (pre) and after (post) a contextual fear conditioning protocol (CFC). During training, seven animals underwent standard CFC (shock group), whereas six (sham group) underwent the same experimental steps, except for the foot-shocks during training. Both groups were tested for CFC 24 h after training. Inset: The shock group shows significant fear behavior compared to before training and to the sham group. Average freezing across animals for each group during testing (sham *n* = 6: training vs test, *p* = 0.63 and shock *n* = 7: training vs test, *p* = 0.02; Wilcoxon signed rank test. Testing: sham *n* = 6 vs shock *n* = 7: *p* = 0.001; Wilcoxon rank-sum test). (**b**) Sleep architecture throughout pre and post recordings averaged across animals and spanning 4 behavioral states: wake (WK), slow wave sleep (SWS), intermediate sleep (IS), and rapid eye movement (REM) sleep. Data expressed as the percentage (%) of each state per 30 s epochs, 15 s overlap. As expected, animals from the shock group remained awake for the majority of time during the first hours after fear training (time awake during post; 1st: 83.8 ± 2.8%, 2nd: 49.2 ± 9.2%, and 3rd hour: 37.3 ± 7.7%), although the exposure to novelty without foot-shock elicited a similar trend in the sham group (1st: 72.6 ± 7.1%, 2nd: 55.2 ± 8.4%, and 3rd hour: 42.3 ± 10.9%). (**c**) REM sleep is not significantly reduced after training. Percentage of REM sleep within each period (pre or post) for groups sham (top) and shock (bottom) (pre × post: sham *n* = 6, *p* = 0.15; shock *n* = 7: *p* = 0.58; Wilcoxon signed rank test). *p < 0.05. Graphs show mean ± SEM (lines/bars ± shades/lines).

We began by chronically recording local field potentials (LFP) from the HPC (dorsal CA1) and RSC (granular and agranular) of freely behaving rats for 3 h before (pre) and after (post) a CFC training, with a memory test 24 h later. During training, animals were divided into two groups (sham, n = 6 and shock, n = 7) (Fig. 1a). The shock group was effectively trained, whereas animals from the sham group were placed in the conditioning chamber during training but did not receive any foot-shock (see “Methods”). During testing, animals from the shock group expressed the fear behavior (i.e., freezing), while animals from the sham group behaved similarly as before training (Fig. 1a, inset).

We then analyzed behavioral states before and after training, with a focus on REM sleep. Before training, the sorting of behavioral states was in line with the rat’s higher propensity to sleep during the light phase (Table 1 and Fig. 1b, left). Although fear training may bias behavioral states towards waking, the overall prevalence of REM sleep was not significantly changed after fear training in our dataset (Table 1 and Fig. 1b,c). Additionally, there was no significant difference in the number of REM sleep episodes between pre and post periods (sham, *n* = *6* per group: *p* = 0.19; shock, *n* = *7* per group: *p* = 0.67; Wilcoxon signed rank test; Table 1).

**Table 1:** Sleep architecture.

State	SWS	IS	REM sleep	WK
**Sham (pre/post)**				
Duration (%)	47.6 ± 6.4/34.3 ± 3.6	2.7 ± 0.5/2.1 ± 0.3	14.5 ± 2.8/6.3 ± 2.1	35.2 ± 9.2/57.3 ± 4.6
# of episodes	79.5 ± 8.6/59.5 ± 10.8	15.3 ± 2.9/11.5 ± 0.9	11.8 ± 2.6/7.2 ± 1.5	145.2 ± 10.7/128 ± 26.8
**Shock (pre/post)**				
Duration (%)	42.7 ± 5.0/32.9 ± 3.5	2.2 ± 0.4/1.6 ± 0.4	8.2 ± 2.3/6.6 ± 1.0	46.9 ± 7.3/58.9 ± 4.0
# of episodes	71.6 ± 11.3/74.14 ± 10.2	12.0 ± 2.0/9.0 ± 2.2	9.3 ± 2.0/7.1 ± 1.8	138.4 ± 33.8/137.1 ± 12.0

Results represent mean ± SEM.

The LFP analysis revealed the classical patterns of delta oscillations (0.5–4 Hz) during SWS, as opposed to the conspicuous theta oscillations (5–10 Hz) during waking (WK), intermediate sleep (IS), and most strongly during REM sleep (Supplementary Fig. S1a). No statistically significant difference was detected in theta relative power during REM sleep from pre to post CFC or between groups in the HPC or RSC channels (Supplementary Fig. S1b,c). However, theta peak frequency during REM sleep significantly increased in the RSC channel from pre to post CFC only in the shock group (Supplementary Fig. S1b). The strength of the phase–amplitude coupling within and between HPC and RSC during REM sleep^16,21^ did not differ between conditions (Supplementary Fig. S2).

### The change in Granger causality (GC) estimation during REM sleep is related to fear memory expression

**Figure 2:**
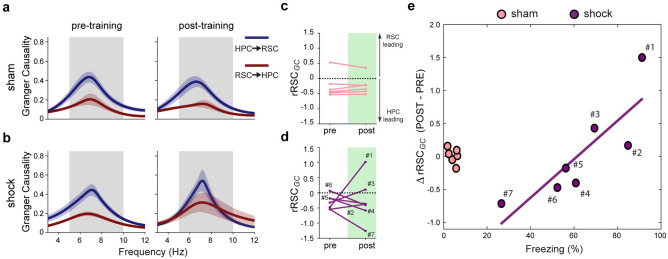
Change in Granger causality (GC) estimates during REM sleep is related to fear memory expression. GC between HPC and RSC channels of rats from sham (**a**) and shock (**b**) groups during REM sleep pre- and post-CFC training. Data plotted in the frequency domain ranging from 3 to 12 Hz. Grey shade, theta range (5–10 Hz). (**c**,**d**) Relative RSC GC (*rRSC*_*GC*_) during REM sleep from pre to post periods. Data points are the difference on GC peak values within theta band between the RSC → HPC and HPC → RSC directions (i.e., *rRSC*_*GC*_ = RSC → HPC—HPC → RSC). Black dashed line: no dominance/threshold for dominance inversion. Note that most of the animals show HPC dominance over RSC theta oscillation during pre for both sham (**c**) and shock (**d**) groups. Numbers represent animals from the shock group sorted from the highest to the lowest freezing during testing (# 1 to # 7). (**e**) Change in *rRSC*_*GC*_ in the shock group (data points from d) from pre to post, defined as ∆*rRSC*_*GC*_, is positively correlated with freezing behavior assessed 24 h after CFC training (*n* = *7*; *R*^2^ = 0.71; *ρ* = 0.93, *p* = 0.007; Spearman correlation). Data points represent the modulation of the strength of RSC → HPC dominance over the HPC → RSC direction from before to after training (post–pre) for individual rats (see “Methods”). Pink and purple represent sham and shock groups, respectively. Purple line: least-squares linear regression for the shock group data points. Graphs show mean ± SEM (lines ± shades).

We previously showed in basal conditions using mean GC calculation that HPC leads RSC theta during REM sleep^16^. We then asked whether GC between the HPC and the RSC during REM sleep would change in response to the CFC (Fig. 2a,b). Consistent with our previous report using GC calculation^16^, we found that, for most animals and conditions, the HPC leads the RSC theta oscillation (Fig. 2c,d and Supplementary Fig. S3a,b). The GC peak frequency was similar between groups, periods, and directions, and is centered in the theta range (Supplementary Fig. S3c). Exclusively for the shock group, there was a notable change from pre to post in the causality strength between the RSC and the HPC (Fig. 2b).

Because the absolute values of GC are interpreted by comparison between directions, we defined the relative RSC GC (*rRSC*_*GC*_) as a more straightforward quantification of dominance between regions (see “Methods”). For our data set, *rRSC*_*GC*_ > 0 indicates that the RSC leads HPC theta oscillation, while *rRSC*_*GC*_ < 0 means the opposite, i.e., that the HPC leads RSC theta oscillation. To deepen the investigation of the major changes in GC shown in Fig. 2b, we plotted the individual *rRSC*_*GC*_ change for the animals of both groups (Fig. 2c,d). Sham animals showed minimal change in *rRSC*_*GC*_ values from pre to post (Fig. 2c). In contrast, animals from the shock group exhibited clear *rRSC*_*GC*_ modulation (Fig. 2d), even with leadership inversion (zero-crossings, animals *#1, #3,* and *#6*). Because *rRSC*_*GC*_ modulation in the shock group was heterogeneous across animals (increasing or decreasing from pre to post; Fig. 2d), we investigated its relation to memory expression. By comparing the freezing time during test with the *rRSC*_*GC*_ change from pre to post (∆*rRSC*_*GC*_), we discovered that memory retrieval 24 h after CFC training is positively correlated with ∆*rRSC*_*GC*_ for the shock group (Fig. 2e). For the sham group, *rRSC*_*GC*_ was nearly invariant between periods (∆*rRSC*_*GC*_ ≈ 0; Fig. 2e). This result indicates that CFC changes the pattern of exchanged influence between the HPC and the RSC theta oscillation during REM sleep. This pattern is indicated by an increased HPC relative influence over RSC theta from pre to post CFC in low-freezing animals, which gradually reverses as animals with stronger freezing levels are assessed, culminating with increased RSC relative influence over HPC theta (Fig. 2e and Supplementary Fig. S4).

### Grangerogram peaks are informative about memory retrieval and are related to transient theta acceleration

**Figure 3:**
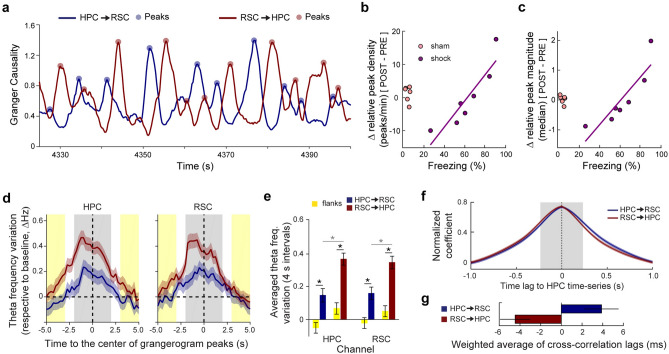
Grangerogram peaks are informative about memory retrieval and are related to theta transient acceleration. (**a**) Smoothed (2-s rectangular kernel) maximum GC values within theta range (grangerogram) during exemplary REM sleep epoch. (**b**,**c**) The changes in relative grangerogram peak density (**b**) and magnitude (**c**) in the shock group from pre to post are positively correlated with freezing behavior during testing (density, *n* = 7: *R*^2^ = 0.82, *ρ* = 0.96, *p* = 0.003; and magnitude, *n* = 7: *R*^2^ = 0.77, *ρ* = 0.96, *p* = 0.003; Spearman correlation). Data points calculated as in Fig. 2e (see “Methods”). Purple line, least-squares linear regression for the shock group. (**d**) Average theta frequency variation across animals/periods in the HPC and RSC channels within time windows centered at grangerogram peaks (4 s, grey shadow). Yellow shadow, 4-s window (2-s each side) used as controls (flanks). (**e**) Theta frequency increases close to grangerogram peaks in the HPC and RSC channels (peaks *n* = 260 vs flanks *n* = 260. HPC channel: HPC → RSC: *t*_(259)_ = − 4.6, *p* = 0; RSC → HPC: *t*_(259)_ = − 6.6, *p* = 0. RSC channel: HPC → RSC: *t*_(259)_ = − 4.3, *p* = 0; RSC → HPC: *t*_(259)_ = − 6.7, *p* = 0), and is even higher in the vicinity of RSC → HPC compared to HPC → RSC peaks (RSC → HPC *n* = 260 vs HPC → RSC *n* = 260; HPC channel: *t*_(518)_ = − 3.9, *p* = 0. RSC channel: *t*_(518)_ = − 3.3, *p* = 10^–4^). (**f**) Average cross-correlation functions between HPC and RSC instantaneous theta frequency in the vicinity of grangerogram peaks (Supplementary Fig. S7a–e). Grey shadow, window that maximizes the difference between directions (± 234 ms; Supplementary Fig. S7h). (**g**) The weighted average of cross-correlation functions in (**f**) shows that the precedence of theta acceleration between regions is dependent on grangerogram direction (HPC → RSC: + 3.9 ± 1.6 ms and RSC → HPC: − 4.5 ± 1.4 ms respective to HPC; HPC → RSC, n = 238; RSC → HPC, n = 239, outliers excluded if outside Mean ± 2 × STD, *t*_(473)_ = 3.8, *p* = 10^–4^; see “Methods” and Supplementary Fig. S7f,g). *p < 0.05. Graphs show mean ± SEM (lines/bars ± shades/lines). Spearman correlation in (**b**,**c**). paired *t* test in [(**e**), comparison with flanks]. unpaired *t* test in [(**e**, between directions) and (**g**)].

In the analysis of GC during total REM sleep for each animal, we detected that all animals expressed significant GC values in both directions (*p* < 0.05, Granger’s F-test, MVGC toolbox). We set out to investigate the dynamics of this interplay between both regions over time by calculating a grangerogram in 3-s duration sliding windows (with 250 ms increment steps; see “Methods” for details). We found that causality is not at all stable, but rather, it represents non-stationary time series that are anti-correlated between directions, with sporadic peaks of intensified causality throughout REM sleep (Fig. 3a; Supplementary Fig. S5). This suggests that the HPC-RSC exchanged influence within REM sleep theta is not a continuous stream, but rather a phasic process (Fig. 3a). By restricting our analysis to the highest peaks of causality (see Methods), we found that, similarly to the results using the GC theta peak value computed for total REM sleep (Supplementary Fig. S3a,b), the HPC → RSC direction shows a trend to supersede the RSC → HPC direction when the density and the magnitude of grangerogram peaks averaged across animals/periods were assessed (Table 2). Although differences were not significant in paired comparisons for density (HPC → RSC × RSC → HPC: pre-sham, p = 0.44; pre-shock, p = 0.23; post-sham, p = 0.44; post-shock, p = 0.48; Wilcoxon signed rank test) or magnitude (HPC → RSC × RSC → HPC: pre-sham, p = 0.43; pre-shock, p = 0.30; post-sham, p = 0.43; post-shock, p = 0.30; Wilcoxon signed rank test). Further, we discovered that relative grangerogram peak density and magnitude also correlated with fear memory expression in the shock group (Fig. 3b,c).

**Table 2:** Grangerogram statistics**.**

Mean ± SEM	Peak of cross-correlograms between directions		Peak density in peaks/min (HPC → RSC/RSC → HPC		Median of peak magnitude (HPC → RSC/RSC → HPC)	
	Pre	Post	Pre	Post	Pre	Post
Sham	− 0.29 ± 0.05	− 0.25 ± 0.04	11.9 ± 1.8/7.8 ± 1.8	11.2 ± 1.4/8.3 ± 1.4	1.1 ± 0.1/0.8 ± 0.2	1.1 ± 0.1/0.8 ± 0.1
Shock	− 0.36 ± 0.04	− 0.39 ± 0.05	11.9 ± 1.7/8.3 ± 0.9	13.2 ± 2.5/9.8 ± 1.9	1.2 ± 0.2/0.8 ± 0.1	1.3 ± 0.2/1.0 ± 0.2

We then narrowed our analysis to the ten highest grangerogram peaks in each direction for all animals and periods (260 peaks for each direction). We observed that theta frequency within both the HPC and RSC was faster in the vicinity of grangerogram highest peaks, as compared to flanking time windows (two 2-s epochs, from − 5 to − 3 s and from + 3 to + 5 s; zero at the peak of the grangerogram; Fig. 3d,e). Interestingly, theta acceleration depended on the direction of the grangerogram peak, as the RSC → HPC direction consistently yielded stronger theta frequency acceleration than the HPC → RSC direction (Fig. 3d,e). Also, although the theta phase modulation of high-frequency oscillations amplitude was not significantly different between groups or after CFC when considering the total REM sleep (Supplementary Fig. S2a,b), the comodulation during grangerogram peaks was much stronger than in the vicinity of data points with minimal causality between directions (see Supplementary Methods; Supplementary Fig. S6a,b). Interestingly, the modulation of RSC fast gamma amplitude by theta phase is significantly stronger in the vicinity of RSC → HPC grangerogram peaks compared to the opposite direction only in the shock group during post (Supplementary Fig. S6c). In the vicinity of the same peaks, HPC fast gamma amplitude modulation by theta phase also increases and becomes significantly stronger in post CFC compared with the pre condition (Supplementary Fig. S6c).

Next, we investigated whether the HPC theta oscillation accelerates first when the HPC leads the RSC theta oscillation (highest HPC → RSC grangerogram peaks), and conversely, whether RSC theta acceleration precedes the HPC when RSC is leading (highest RSC → HPC grangerogram peaks). We addressed this question by first performing the wavelet spectral decomposition of the HPC and the RSC LFP time series from − 2 to + 2 s centered at the highest grangerogram peaks (as above, see Methods for details; Supplementary Fig. S7a–b). Then, we computed the cross-correlation function between the instantaneous HPC and RSC theta frequency time series for each one of the selected grangerogram peaks (Fig. 3f, Supplementary Fig. S7c–g). Such analysis showed that HPC theta was biased to accelerate before RSC theta during the highest HPC → RSC grangerogram peaks. In contrast, the opposite occurred during the highest RSC → HPC grangerogram peaks (Fig. 3g). Altogether, these results suggest that grangerogram peaks may represent increased HPC and RSC interaction during REM sleep and such communication is marked by a transient acceleration of the theta oscillation.

### Phasic REM events are related to transient modulation of Granger causality

**Figure 4:**
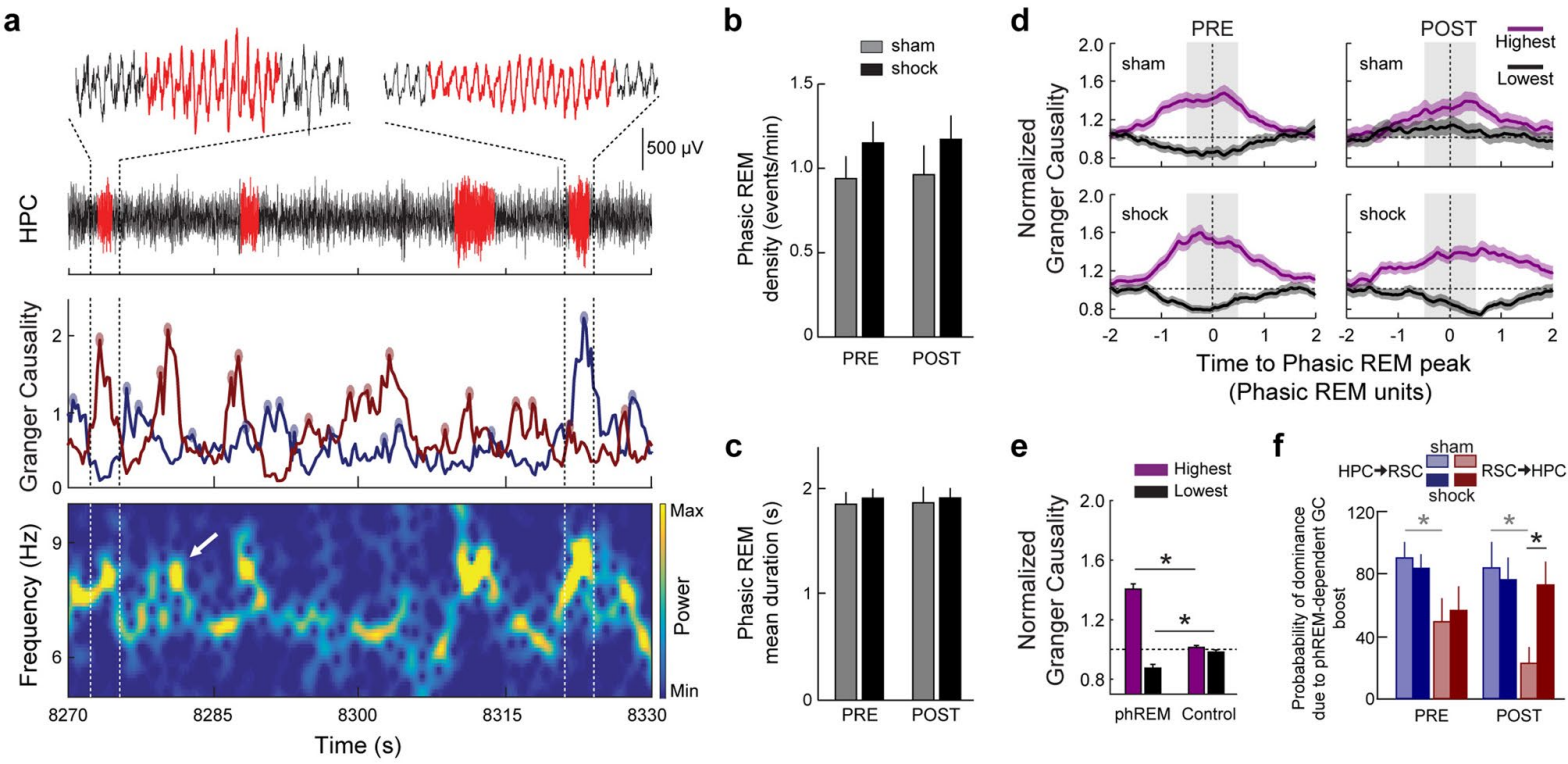
Phasic REM events are related to transient modulation of granger causality. (**a**) Phasic REM (phREM) events in relation to the simultaneous grangerogram time series and HPC spectrogram. Top, HPC LFP raw trace in black, phREM events in red. Insets, zoom in (3-s window) on representative phREM events in which the RSC leads HPC theta (left) and HPC leads RSC theta (right). Grey dashed lines highlight insets across all plots. Middle, grangerogram time series and their selected peaks (dots). Bottom, HPC spectrogram showing the characteristic transient theta acceleration related to phREM events. White arrow, exemplary transient theta acceleration synchronous with a grangerogram peak, which was not detected as a phREM event. (**b**,**c**) No difference was found in phREM mean density among groups or periods (Sham, *n* = 6, Shock, *n* = 7; pre vs post: sham, p = 0.56; shock, p = 0.94. sham *vs* shock: pre, p = 0.29; post, p = 0.36; (**b**) or duration (Sham, *n* = 6, Shock, *n* = 7; pre vs post: sham, p = 0.32; shock, p = 0.68. sham *vs* shock: pre, p = 0.62; post, p = 0.52; (**c**). (**d**) Grangerograms centered at phREM events, normalized by phREM length (x-axis), by each respective baseline grangerogram (y-axis), and averaged across groups/periods. Plot shows the difference in GC modulation between the directions with highest and the lowest values (see “Methods”). Black dashed lines: vertical, center of phREM events; horizontal, baseline. (**e**) The GC averaged across one phREM unit (**d**, grey shadow) is significantly upmodulated in the highest direction and downmodulated in the lowest direction as compared with controls centered at random data points (*n* = 396/group. Highest: phREM × control, *t*_(790)_ = 10.8, *p* = 10^–25^; Lowest: phREM × control, *t*_(790)_ = − 4.0, *p* = 10^–5^; unpaired *t* test). Black dashed line, baseline. (**f**) The likelihood that the HPC will lead the RSC theta oscillation due to phREM-dependent GC upmodulation shows a trend to be higher than the opposite direction for both groups, although only reaching significance for the sham group (Sham, *n* = 6, Shock, *n* = 7; HPC → RSC vs RSC → HPC: pre-sham, p = 0.04; pre-shock, p = 0.23; post-sham, p = 0.006; post-shock, p = 0.87; Wilcoxon signed rank test). No significant differences was found between groups, except in the RSC → HPC direction during post, in which the probability of group shock is higher than sham (Sham, *n* = 6, Shock, *n* = 7; sham vs shock: HPC → RSC-pre, p = 0.26; RSC → HPC-pre, p = 0.90; HPC → RSC-post, p = 0.63; RSC → HPC-post, p = 0.04; Wilcoxon rank-sum test). *p < 0.05. Graphs show mean ± SEM (lines/bars ± shades/lines).

Since it has been shown that theta acceleration and high frequency oscillations (40–120 Hz) boost occur during phasic REM (phREM) sleep^14^, we examined whether changes in directionality occur during phREM. Consistent with previous results^14,15^, on average, 3.33% of the total REM sleep duration was classified as phREM sleep. The phREM events lasted 1.88 ± 0.05 s and showed an average density of 1.06 ± 0.07 events/min (mean ± SEM; *n* = 396 phREM events from *N* = *13* animals during pre and post periods; Fig. 4a). No significant difference between groups or periods was observed (Fig. 4b,c). In addition, as shown in Fig. 4a (top, four events within 1 min), the phREM events are not periodic and seem to be unevenly distributed across REM sleep epochs (80% or more of the phREM events occur within only 31.3% of REM epochs in average across animals/periods).

We observed that phREM events seem to be related to GC values fluctuation in both directions (Supplementary Fig. S8a and Fig. 4a). To quantitatively investigate how phREM events are linked with grangerogram modulation irrespective of direction, we normalized their duration and determined whether periods with high GC were positively linked with phREM. We found out that phREM is indeed associated with higher GC values (Fig. 4d). By integrating the normalized GC values during phREM events (gray shaded area in Fig. 4d), we were able to show that GC transiently increases significantly in the highest direction (the one with the stronger causality) while it decreases in the lowest direction, as compared to windows centered at random data points within the same REM sleep epoch (control) (Fig. 4e). Importantly, this effect was not observed when the RSC LFP was surrogated by a simulated time series projected using the LFP from the HPC as the input of an autoregressive model (Supplementary Fig. S9; see “Methods”). This indicates that transient theta acceleration during phREM events by itself does not induce spurious GC modulation.

To test whether the modulation of GC specifically during phREM is sufficient to invert GC directionality, we calculated the conditional probability of HPC → RSC and RSC → HPC dominance during phREM events (e.g., considering only phREM events in which the HPC → RSC direction was significantly upmodulated, the probability that the GC in the HPC → RSC direction will be stronger than the RSC → HPC direction). Consistent with our’s (Fig. 2a,b and Supplementary Fig. S3a,b) and previous results^16^, we found that, on average, the HPC → RSC direction tends to supersede the converse direction in both groups during the pre-training period (Fig. 4f). Conversely, the RSC → HPC direction shows a higher probability to lead in the post-training period, specifically in animals from the shock group (Fig. 4f). Taken together, these findings suggest that the increased influence of the RSC over the HPC during phREM plays a role in memory consolidation of CFC.

## Discussion

We used a statistical tool tuned to infer causality between time series—the Granger causality (GC)^19,20^—to investigate the exchanged influence between two brain regions relevant for memory processing after CFC training: the HPC and the RSC. During REM sleep that follows CFC training, we observed a modulation of the relative influences the HPC and RSC exert upon each other’s theta oscillation, in a manner that is significantly related to learning performance. The interplay detected between the HPC and the RSC is strengthened during transient time windows which are synchronized with theta acceleration epochs in both regions, in close relation to phasic REM sleep. Our results support the notion of a conspicuous neurophysiological relationship between the HPC and the RSC during REM sleep.

Since most of our results rely on GC estimates, it is essential to discuss the reliability of GC values in the face of potential confounds such as volume conduction, signal stationarity, the use of small moving windows, and the possibility of a common input, which together could lead to erroneous causality estimates and misleading interpretations^22^. The first important issue is the possibility of volume conduction of theta oscillation from the HPC to the RSC during REM sleep^23^, which may jeopardize GC estimates. While some volume conduction may exist, the coupling of RSC neuronal activity to hippocampal theta oscillation, if not evidence of local generation, suggests that synaptic input can drive membrane conductance of target neurons. In this case, synaptic activity would entrain local theta generator within the RSC^16,18,23^. Since there is no optimal tool tuned to rule out the specific effect of volume conduction on causality estimates^19,20,22^, we mitigated this confound through a rigorous selection of representative electrodes for both regions (the criteria comprised histological, oscillatory, and comodulation patterns; see “Methods”). Also, we used two controls for the comparison of causality estimates within (pre vs post) and between (sham vs shock) subjects. An important new piece of evidence provided here is the time lag difference in theta acceleration between HPC and RSC in the vicinity of grangerogram peaks (Fig. 3f,g), which makes it quite unlikely that volume conduction from the HPC is the only effect responsible for RSC’s theta oscillation. Indeed, the RSC theta frequency tends to accelerate before the HPC in the vicinity of RSC → HPC grangerogram peaks. Importantly, the analysis of two time-series with dynamic changes in the causality relationships between directions, may elicit asymmetric cross-correlation functions^24^, preventing a clear definition of the time lag between them. To investigate this trend, we have not used the peak but the weighted average of the cross-correlation functions to quantify their asymmetry. Thus, the specific values of time lag difference of the RSC channel respective to the HPC (+ 3.9 ± 1.6 ms in the HPC → RSC direction and − 4.5 ± 1.4 ms in the RSC → HPC direction) reported here should not be taken as functionally relevant. Further investigation on this matter would be necessary to clarify the specific inputs driving this communication and the real time lags of theta acceleration between the HPC and the RSC.

To minimize the effect of non-stationarity, in addition to choosing electrodes with constant means across data, each sample data (15-s chunks for all REM and 3-s chunks for grangerograms) was detrended and z-scored before computing GC estimates. This reduced the contribution of very slow fluctuations (DC-like) and of amplitude asymmetry between animals (resulting from varied electrode impedance) on GC calculations.

Since small window sizes may also hazard GC estimates^25^, in addition to testing the significance of our grangerogram calculation within 3-s moving windows using Granger’s F test^19^, we further checked the amount of false positive values by computing grangerograms using a surrogated dataset (see “Methods”). This analysis elicited an average of 5.41% of significant GC values for at least one direction across animals/periods. In contrast, 97.7% of GC values were deemed significant in the original dataset.

Another caveat worth considering is the possibility that a third-party or more nodes outside of this hypothetical binary network exert influence on HPC/RSC theta interaction during REM sleep. The selective inhibition of GABAergic neurons from the medial septum (MS) after training in HPC-dependent memory tasks dramatically decreases HPC theta oscillation as well as memory performance^11^. The MS is thought to pace HPC theta rhythm mainly by the inhibitory drive of parvalbumin positive (PV+) cells upon other PV+ interneurons within the HPC^26^. These cells in the MS typically burst in a theta range pace^27^ and also project to RSC interneurons^28^. Furthermore, it was recently shown that PV+ interneurons in the cortex are more active during REM sleep, especially the subset which was active during previous waking periods^29^. These results suggest that theta oscillations within the HPC and the RSC may arise from a similar mechanism dependent on the MS, although many other regions may participate, as for instance the supramammillary nucleus^30^, the mammillary bodies^21^, or the claustrum, whose projections activate the dentate gyrus in the HPC and the RSC during REM sleep^31^. The anterior thalamic nuclei (ATN) are also strong candidates to participate in this network since they are bidirectionally connected with both the HPC and the RSC, show similar theta oscillations, and have been related to the processing of mnemonic spatial information^32^. Until all these regions are simultaneously studied using, for instance, conditional causality analysis^19,25^, any evidence of theta driven exchanged influence between the HPC and the RSC should be interpreted carefully.

Irrespective of the structures involved in memory consolidation, several lines of evidence reinforce the importance of correlated activation of HPC and RSC for the processing of waking experiences. Both regions are, individually and independently, important for memory encoding and consolidation during early or late time windows after training^1^. They also project to each other mutually^33^. During waking, electrophysiological events as theta, gamma and fast-gamma oscillations, as well as sharp-wave ripple complexes, are coordinated and phase-locked between the HPC and the RSC^18^. Theta coherence between the HPC and the RSC increases during the encoding and retrieval of emotional memory^17^ and the interplay between the HPC and the RSC is relevant for memory persistence^2^. Interestingly, during REM sleep, HPC theta modulation of gamma oscillations and unitary activity within the RSC seems to be even stronger than during waking^16,21^, and this coupling during retrieval was shown to be related to behavioral performance^34^. These observations raise the hypothesis that HPC-RSC communication comprises a key phenomenon of spatial memory processing^3^, which may rely on REM sleep-dependent activity^5,35^.

Our results indicate that theta oscillation during early post-training REM sleep, shown to be relevant for memory consolidation^11^, may support the HPC-RSC functional interplay. Accordingly, theta coherence among basolateral amygdala, HPC, and medial prefrontal cortex during REM sleep correlates with the expression of emotional memory^13^. Although the involved mechanisms are still unclear, theta synchronization within the HPC-neocortical network during sleep could promote the reinforcement or weakening of tagged synapses linking distant neuronal units^7,23^. For instance, long-term potentiation or depression can be obtained by inducing neuronal discharge at specific phases of the theta cycle^36^, which ultimately would result in inter-regional synaptic plasticity. Our results also suggest that GC phasic increases during REM sleep underlie modulation both of theta activity and task performance (Fig. 3).

Several new pieces of evidence may help clarify these findings. For instance, it has been recently reported that inhibitory projections from the dorsal CA1 (HPC) and excitatory projections from the ATN through thalamocortical pathways targeting the RSC molecular layer, both connect with apical dendritic tufts of layer V principal neurons and modulate CFC retrieval^37^. Interestingly, this subset of inhibitory projecting cells from the dorsal CA1 layer to the RSC fires rhythmically at a theta pace^38^, and their inhibition improves retrieval, whereas inhibition of the ATN input impairs retrieval^37^. This is in line with our finding that increased GC leadership of the HPC over the RSC theta oscillation is related to poor retrieval (Figs. 2e, 3b,c). Moreover, it was recently shown that late (i.e., 24 h) post-training optogenetic reactivation of the RSC ensemble associated with CFC exclusively during offline states (light anesthesia or sleep), but not during waking, promotes systems consolidation and increases LFP power in the gamma band (~ 100 Hz) in regions related to recent and remote memory expression, i.e., the HPC and the anterior cingulate cortex, respectively^39^.

Another critical aspect of the present results is the role of phREM in HPC-RSC communication during sleep. We observed that GC modulations are episodic and related to phREM events (Figs. 3a,d–g, 4a,d–f). However, the contrasting density of GC peaks (~ 20 peaks/min, both directions; Table 2) compared to phREM events (~ 1 event/min; Fig. 4b) may suggest that the criteria classically used to detect phREM events is too stringent. For instance, Fig. 4a (bottom, white arrow) shows an event with theta acceleration and increased power related to a prominent grangerogram peak, which was not detected as phREM. This underestimation could explain why our results of increased comodulation around the GC peaks (Supplementary Fig. S6) are at odds with previous results, which showed decreased comodulation during phREM events, as compared to tonic REM sleep^15^.

The transient increases in the power and frequency of theta oscillations during REM sleep, which defines phREM events, are known to increase theta- and gamma-bands coordination and neuronal activity within and between the tri-synaptic pathway^14^. Although present during waking^18^, theta coupling of high-frequency oscillations in the HPC and the RSC is more prominent during REM sleep^16,21^. Moreover, the coupling between theta rhythm and high frequency oscillations was shown to fluctuate across REM sleep with transient periods of synchrony between hippocampal and cortical regions^40^. Here, we showed that these transient intervals of stronger REM sleep coupling may take place during periods of boosted HPC-RSC exchanged influence putatively related to memory processing (GC peaks; Supplementary Fig. S6). Convergent with this notion, we also showed that theta-fast gamma coupling in the RSC was stronger in the RSC → HPC compared to the opposite direction only after learning in the shock group, simultaneously to a stronger theta-fast gamma coupling in the HPC compared to before learning (Supplementary Fig. S6c). This suggests that the bidirectional theta-driven modulation of high-frequency oscillations between these regions, especially during phREM, contributes to memory processing.

We showed that these GC peaks in both directions are related to transient theta accelerations in the HPC and the RSC, suggesting their relation to phREM events (Fig. 3d–g). Moreover, by investigating GC during phREM events, we show that GC is indeed modulated irrespective of the direction (Fig. 4d,e). Most interestingly, by restricting the analysis to phREM events in which GC was significantly upmodulated, we show that the probability of the RSC to lead HPC theta during post is higher for the shock group compared to the sham group (Fig. 4f), consistent with the increased theta peak frequency of RSC during REM sleep from pre to post learning only in the shock group (Supplementary Fig. S1b). This result reinforces the notion that the RSC causality upon HPC theta is positively correlated to memory performance, and also supports the involvement of phREM events in spatial memory processing early after training. Altogether, these results suggest that phREM events are intrinsically linked to GC modulation, so as to constitute a privileged window for information processing within and between brain regions relevant for memory. However, the mechanisms involved in the generation of phREM events are still unclear, and, to the best of our knowledge, there is so far no report of a direct relation between phREM and memory processing.

A shred of indirect evidence worth considering is the described temporal relation between phREM events and phasic pontine activity (a.k.a P-waves) in rodents^41^. Similarly to what we found using GC (Fig. 3b,c), the change in the density of P-waves from pre- to post-training REM sleep are correlated to learning and gene expression within the dorsal HPC^42,43^. Rodent P-waves have been described to contribute to theta synchrony between HPC and amygdala during REM sleep^44^, while single and clustered discharges elicit, respectively, ~ 0.2 and ~ 0.4 Hz boosting in dorsal HPC theta frequency during REM sleep^41^ (see also Fig. 3d,e). Future experiments will determine the role of the ascending reticular activating system in the generation of phREM and GC dynamics during REM sleep.

Finally, some key questions emerge from our results. Are phREM events indeed required for systems consolidation during REM sleep? Are the binary GC results between HPC and RSC communication unveiling a direct relationship between these regions, or are there other regions involved? Although these questions need further investigation, the present results provide the first direct evidence that (i) the interplay between the RSC and the HPC during REM sleep is relevant for memory consolidation, and (ii) that this interplay is triggered by transient theta frequency acceleration epochs related to phasic REM events. Altogether, our results support the notion that theta acceleration epochs constitute privileged windows of opportunity for the reprocessing of recently encoded information, with latent outcomes for synaptic plasticity taking place during REM sleep^5,35,43^. Since recent evidence implicates the RSC as a hotspot for oneiric activity^45^, it may also be that mnemonic information processing and dreaming during REM sleep share some fundamental mechanisms.

## Materials and methods

### Animals

Male Sprague–Dawley rats (*n* = 13, 3–4 months old, 250–300 g, RRID: RGD_5508397) supplied by Javier Labs and Charles River Laboratories and individually housed in a well-controlled environment (temperature 22 ± 2 °C and humidity at 75%) under a 12 h light/dark cycle (lights on at 07:00), with food and water ad libitum. The experimental design was approved by the institutional animal care and use committee of the University of Lyon 1 and the French Ministère de l’Enseignement Supérieur et de la Recherche, and were in accordance with the French and European Community guidelines for the use of animals in research, as well as the Ethical Committee on Animal Use of the Federal University of Rio Grande do Norte, Brazil (permit # 025/2014). The study was carried out in compliance with ARRIVE guidelines and sample size was not calculated.

### Multielectrode implantation

Rectangular-shaped arrays of tungsten microelectrodes (35 µm diameter; with ~ 100–350 kΩ impedance at 1 kHz; California Fine Wire) were manufactured and chronically implanted into the dorsal HPC (AP: 3.0–4.4; DV: 3.8; ML: 1.1–2.6 mm) and RSC (AP: 4.5–6.5; DV: 2.2; ML: 1.2–2.9; angle: 13°)^46^ to record Local Field Potentials (LFP), as previously described^16^. Two stainless steel screws were implanted over the right frontal and left parietal cortices for monitoring electrocorticographic activity (ECoG), as well as two additional spherical electrodes placed in the nuchal muscles for electromyography (EMG) recordings. A screw was placed over the cerebellum as a common reference for all electrodes. The animals recovered for at least 7 days after surgery.

### Electrophysiological recordings

Before the beginning of experiments, animals were handled and habituated to the recording chamber for 3 days. For each recording session, rats were plugged after enough handling to keep animals still (~ 2 min). The freely moving animals were recorded continuously throughout the experiment for at least 6 h, except during behavioral conditioning, for which the recording was interrupted but the animal was not disconnected from the headstage cable. The recording was resumed immediately after training. The analysis was focused on the period between 10:00 and 18:00. The electrophysiological recordings were performed using a multichannel acquisition processor (MAP System, Plexon). Signals were pre-amplified (VLSI headstages, 20× gain, Plexon), filtered (0.1–500 Hz), amplified (20–100×), digitized at 1 kHz, and stored for further analysis.

### Behavioral conditioning

The animals were randomly divided into two groups, sham (*n* = 6) and shock (*n* = 7). For a comparative analysis, electrophysiological data were continuously recorded from rats of both groups during 3 h before (pre) and 3 h after (post) the training session in a Contextual Fear Conditioning (CFC) paradigm. Training and testing were performed in a custom-made conditioning chamber (40 × 40 × 60 cm) with a stainless steel grid floor as previously described^35^. The chamber was cleaned with 10% ethanol before each session. Training sessions of the shock group consisted of placing the animal in the conditioning chamber for a 2-min habituation period, followed by two 2 s, 0.7 mA foot-shocks, spaced by 2 min (at minutes 2 and 4). After another 2-min period, animals were retrieved and returned to the recording chamber during the post period (Fig. 1a). Animals from the sham group went through the same protocol but did not receive any foot-shock. Animals with no reaction to shock and freezing time smaller than 10% during testing were assigned to the sham group. Animals from both groups were placed in the same conditioning chamber for 2 min 24 h later in the absence of foot-shocks (testing session). Freezing behavior (defined as the complete absence of movements except for breathing) was automatically quantified as the percentage of time animals froze during pre-training (2 min) and testing (2 min), using a video actimetry-based paradigm with a side view^35^.

### Data analysis

LFPs were analyzed using customized scripts running in Matlab^®^ (MathWorks). Five-second epochs of electrophysiological data (LFP, ECoG, and EMG) were used to sort behavioral states into 4 categories: waking (WK), slow wave sleep (SWS), intermediate sleep (IS), and rapid eye movement (REM) sleep, according to standard criteria^16^ (Fig. 1b). Behavioral states were classified by two experienced researchers and all further analyses were performed on state-tagged data epochs lasting a minimum of 15 s. For each 5-s period within epochs, the power spectral density (PSD) of LFP signals were calculated using the Welch’s method (*pwelch* function, 1-s windows, no overlap), and normalized as a percentage of each frequency relative to the total power. We calculated the mean relative power by averaging each 5-s period for each state for each animal. Grand averages were calculated averaging all animals according to the behavioral state. Since subsequent analyses were executed exclusively on REM sleep periods, one representative channel for each region (HPC and RSC) was chosen based on the following criteria: (i) a classical profile of relative power within specific frequency bands (delta: 0.5–4 Hz, theta: 5–10 Hz) for each behavioral state; (ii) strong theta oscillations during REM sleep compared among all channels; and (iii) strong phase-amplitude coupling during REM sleep, defined as high-frequency oscillations (gamma: 40–100 Hz and fast gamma: 100–160 Hz) amplitude modulation by the theta phase to a minimum intensity of 1 × 10^–3^, well above what has been considered significant^15,43^. In addition to these criteria, the representative HPC and RSC channels for each animal were chosen by histological confirmation of electrode positioning in the pyramidal layer of the dorsal CA1 in the HPC and deep layers of the RSC (Supplementary Fig. S10). HPC channels went through a second confirmation to check for the phase-amplitude coupling profile specific of the pyramidal layer (Supplementary Fig. S2a). For statistical comparison, the peak frequency and relative power within theta band for channels of each animal were defined respectively as the absolute frequency with maximum power in the normalized PSD and the total power summed across the theta band (5–10 Hz) normalized by the total power of all frequencies (Supplementary Fig. S1).

### Granger causality analysis

As previously described^19^, the Wiener–Granger causality (GC) method was used to determine the directionality and strength of the exchanged influence between LFP signals recorded from electrodes located in the HPC and the RSC during REM sleep^16^. This method determines to which extent one time series improves the prediction of another time series, in comparison with the prediction obtained using solely its own past values. GC was computed on the selected HPC and RSC LFP channels using the multivariate GC (MVGC) toolbox^19^ blindly to the freezing time. This toolbox is optimized to produce reliable inference on the exchanged influence between simultaneously recorded time series in the frequency domain, using vector autoregressive models. The input data were z-scored LFP from both regions decimated to 200 Hz and organized as 15-s chunks. No filtering was performed because it potentially compromises causalities estimates^19^. The model order, i.e. how many data points of the past are used to predict the present values of a time series, was calculated using the Levinson–Wiggins–Robinson (LWR) algorithm for the regression mode, as it is default for the MVGC toolbox, associated with the Akaike information criteria^19^. A single model order was defined across data sets (animals/periods) using a heuristic approach. Because the computed model order varies with the size of the time series, we separated the decimated LFP (HPC and RSC) from REM sleep periods of all animals into chunks of sizes ranging from 5 to 100 s. Small chunks were created by subdividing REM episodes. Longer chunks were produced from longer REM episodes only, without the concatenation of state-specific episodes. Chunks of data were arranged as trials of a given animal/period and were used to calculate the model order for different chunk lengths. We found that chunk lengths higher than 60 s for all animals/periods narrowed to a model order of 25 data points (Supplementary Fig. S11). This model order value was used for all the subsequent GC analysis. The computation of GC for a pair of time series elicits two values (one for each direction). However, the absolute GC value is not sufficient to determine the dominance of one time series upon the other. We then defined the relative RSC GC ($rRSC_{GC}$) as the subtraction of the GC peak value within theta band in the HPC → RSC direction from the RSC → HPC, as a measure of the leadership in the interplay between both regions:

1 $$rRSC_{GC} = GC_{(RSC \to HPC)} - GC_{(HPC \to RSC)} .$$

In our data set, if RSC theta information helps predict HPC theta better than HPC theta information helps predict RSC theta, $rRSC_{GC}$ > 0 and we say that the RSC leads the HPC theta. If $rRSC_{GC}$ < 0, we say that the HPC leads the RSC. We also performed a grangerogram analysis by computing the GC in a 3-s moving window with 250 ms steps. This window length was used because it was the minimum time interval that elicited more than 95% (97.7% average, minimum of 89%) of significant GC values for at least one direction among windows for each animal/period, as computed using Granger’s F-test (*p* < 0.05, with multiple hypothesis test correction based on a false discovery rate—MVGC toolbox)^19^. Non-significant windows were excluded from subsequent analyses. To further check the reliability of grangerogram values, we computed grangerograms inputting the observed HPC time series with the flipped RSC time series from the end to the beginning of each REM sleep episode. This calculation using the surrogated RSC time series elicited only 5.41 ± 0.52% (mean ± SEM) of significant GC values for at least one direction across animals/periods. We also defined peaks of expressive GC across the grangerograms (see Fig. 3a). Candidate GC peaks were determined as the positive-to-negative zero-crossings of the derivative of grangerograms. Because the grangerogram is not stationary and we wanted to detect only relevant transient GC increases, candidate GC peaks were narrowed down to those displaying a 0.3 minimum prominence. Prominence was determined as the GC absolute difference between a given peak and its deepest neighbor trough within a time window limited bilaterally by the closest peak higher than the given peak or the left/right end of the REM sleep episode. Since we found that theta acceleration is related to GC modulation, we also checked if the acceleration itself would yield higher GC values as a spurious result of GC computation. Because the MVGC toolbox is based on vector autoregressive models and to ensure naturalistic theta acceleration during the same windows of phasic REM events, we used the HPC time series to generate a simultaneous autoregressive RSC time series, based on the following sequential Eqs. (2, 3, and 4). We first projected a simulated RSC LFP time series in relation to the past values of the HPC time series:

2 $$LFP_{{RSC_{(t)} }} = P \times {(0.57 \times LFP_{HPC} } _{(t - 1)} + 0.28 \times LFP_{{HPC_{(t - 2)} }} + {0.14 \times LFP_{HPC} }_{(t - 3)}),$$

where *t* is in milliseconds (ms) and *P* is the ratio between the average power of RSC and HPC theta rhythm for each animal/period. This was intended to make the RSC simulated time series to have a similar power as the original one. We then added a white gaussian noise (*ε*) with half the power of *LFP*_*RSC*_ in (2):

3 $$LFP_{{RSC_{(t)} }} = LFP_{{RSC_{(t)} }} + \varepsilon ;$$

finally, we added the *LFP*_*RSC*_ simulated time series (3) with information from its own past values:

4 $$LFP_{{RSC_{(t)} }} = 0.67 \times LFP_{{RSC_{(t)} }} + 0.19 \times LFP_{{RSC_{(t - 1)} }} + 0.095 \times LFP_{{RSC_{(t - 2)} }} + 0.048 \times LFP_{{RSC_{(t - 3)} }}$$

As expected, our simulated data elicited significant GC values in the theta range only for the HPC → RSC direction when computed for the total REM sleep for each animal/period (Granger’s F Test). By computing the grangerogram using the HPC real and RSC simulated time series during phasic REM events (see below), we showed that theta acceleration itself does not induce transient GC modulation (Supplementary Fig. S9).

### Theta frequency variation analysis

For the calculation of theta frequency variation in the vicinity of grangerogram peaks (Fig. 3d,e), we first calculated the spectrogram using the short-time Fourier transform restricted to the theta band (5–10 Hz, 0.1 Hz bandwidth) in the same windows in which the grangerograms were calculated (3 s windows, 0.25 s steps). Next, the peak frequency for each data point was detected from − 5 to + 5 s centered at the grangerogram peaks. We then determined a baseline frequency time series as the average of the time series in the neighborhood of 1000 random data points for each animal/period (pre or post). Finally, we defined theta frequency variation by the subtraction of the baseline time series from each peak frequency time series. To determine the temporal difference of theta acceleration between the HPC and the RSC channels in the vicinity of grangerogram peaks, we went through the following steps (Supplementary Fig. S7): (1) first, we performed the Morlet wavelet spectral decomposition (scaled pseudo-frequencies of 5–10 Hz, 0.1 Hz bandwidth) on HPC and RSC chunks of LFP time series spanning ± 2 s in relation to grangerogram peaks (4000 time points from each region’s time series; Supplementary Fig. S7a,b). (2) we then detected the frequency with maximum power for each time point to extract the instantaneous frequency time series for each region (one 4000-data points vector for each region; Supplementary Fig. S7c,d). (3) Next, we computed the cross-correlation between vectors of instantaneous frequency time series from both regions (Supplementary Fig. S7e), then (4) we used the normalized cross-correlation coefficient related to each time lag as a weight (Supplementary Fig. S7f), and we computed the weighted time lag average for each cross-correlation vector (Supplementary Fig. S7g). These weighted average values depict the asymmetry seen in the cross-correlation functions, indicating a trend of precedence of a given time series in relation to the other.

### Phasic REM detection

Phasic REM events (phREM) were detected as previously described^14^. First, the LFP of HPC channels of each REM sleep episode was filtered within the theta range (5–12 Hz; *eegfilt* function). The peaks of the theta cycle were retrieved by the detection of positive-to-negative zero-crossings of the derivative of the filtered data. The inter-peak interval was smoothed with an 11-sample rectangular kernel. Candidate phREM events were defined as those subsets of continuous data points below the 10th percentile of the smoothed inter-peak interval distribution. Finally, phREM events were retrieved whenever candidate phREM events met all following heuristic criteria: (1) duration longer than 900 ms; (2) minimum inter-peak interval within the candidate phREM shorter than the 5th percentile of smoothed inter-peak intervals; and (3) mean amplitude within theta band of the candidate phREM higher than the mean theta amplitude of all REM sleep. Consistent with previous results^13,14^, an average 3.33% of all REM sleep recorded were deemed as phREM across animals/periods. We also computed an average grangerogram time series in windows centered at phREM events. To that aim we normalized phREM events by their respective lengths prior to getting the grangerogram time series centered at them. A linearly spaced vector of 303 data points was created and GC values within − 2 to + 2 phREM units were interpolated across these points. For the determination of GC modulation by phREM, we first computed a baseline grangerogram for each direction by getting the average of 100,000 random grangerogram time series with the same 303 data points. GC modulation was determined by the ratio between the grangerogram related to each phREM event and its respective baseline (animal/period/direction wise). These modulations were classified as positive or negative based on the average modulation across the data points within the normalized phREM event (− 0.5 to + 0.5 phREM units), as compared to baseline. The average baseline modulation distribution was computed using also the 100,000 random time series described above. Positive modulation was defined as the average grangerogram modulation higher than the 60th percentile of the distribution of average baseline modulation, whereas negative modulation was defined as those below the 40th percentile.

### Histology

At the end of experiments, rats were euthanized with pentobarbital (100 mg/kg, i.p.) and transcardially perfused through the left ventricle with ringer lactate, followed by 4% paraformaldehyde solution at 4 °C. The brains were removed and stored sequentially at 4 °C in 4% paraformaldehyde and 30% sucrose for at least 24 h each treatment; then frozen after been frontally sectioned at 30 µm in a cryostat (Microm). The positions of the electrode tips were determined based on a rat brain atlas after examination of the brain sections counterstained with neutral red^46^.

### Statistical analysis

The behavioral and electrophysiological data, when normally distributed (Kolmogorov–Smirnov test), were analyzed using two-tailed two-way analyses of variance (ANOVA), with Tukey–Kramer test for multiple comparisons or two-tailed paired and unpaired *t* tests for comparisons between pairs of data with a bootstrap strategy (10,000 resamplings) to control for multiple comparisons. For data where normality was not observed, we used Wilcoxon signed rank test and Wilcoxon rank-sum test for paired and unpaired comparisons, respectively; with a bootstrap strategy (10,000 resamplings) to control for multiple comparisons where appropriate. The Spearman’s correlation coefficient (*ρ*) was used to determine the ranked dependency between two variables, and a p value was determined for each correlation using the distribution of *ρ* for all possible permutations of values within compared variables. For each correlation, we also computed a least-squares linear regression and the coefficient of determination (R^2^) to quantify how well the linear fit model explained the distribution of data points. For all analyses, significance was set at 5%. The data are reported as mean ± SEM.

## Supplementary Information

Supplementary Information.

## Data Availability

The electrophysiological recordings and behavioral data that support the findings of this study are available from the corresponding authors upon request. The customized codes for analysis and control simulations were written in Matlab^®^ and are also available from the corresponding author upon request.
