# Supplementary material for: Hippocampus-retrosplenial cortex interaction is increased during phasic REM and contributes to memory consolidation
Source: Sci Rep. 2021 Jun 22;11:13078. doi: 10.1038/s41598-021-91659-5 (PMC8219679; doi:10.1038/s41598-021-91659-5)
Supplement: Supplementary file 1 — Supplementary Information. [file 41598_2021_91659_MOESM1_ESM.doc]

Supplementary Materials

***Supplementary Materials and Methods***

*Phase–amplitude coupling*

The modulation index (MI) was computed to quantify the phase–amplitude coupling between different frequency oscillations . The MI is defined as the normalized Kullback–Leibler distance of a uniform distribution from the mean amplitude distribution of a high-frequency oscillation across all phases of a simultaneous low-frequency oscillation. Phase and amplitude information were obtained from signals recorded from the previously selected HPC and RSC channels. Low-frequency oscillations were restricted to the theta band range (5 – 10 Hz), whereas high-frequency oscillations were subdivided into gamma (40–100 Hz) and fast gamma (100–160 Hz) frequency bands. Comodulogram maps were produced by calculating the MI between the phase of frequencies ranging from 5 to 10 Hz (0.5 Hz bandwidth) and amplitudes ranging from 40 to 160 Hz (5 Hz bandwidth). No overlap was used for phase or amplitude. The LFP data were filtered using a linear finite impulse response filter coded in the *eegfilt* Matlab® function from the EEGLAB toolbox. Phases and amplitudes of filtered data were obtained using the Hilbert transform. We used 18 phase equal bins ranging from -π to +π radians to project the distribution of mean amplitudes per phase bin. For the analysis of comodulation across the whole amount of REM sleep recorded for each animal/period, a threshold was computed to define significant MI values by shuffling REM epochs (5-s windows) of the amplitude time series of a given high-frequency oscillation respective to the phase time series, prior to computing comodulogram maps. Significant MI values were defined as those above the 75^th^ percentile of the distribution of the maximum values of each shuffling-elicited comodulogram map, summed with its interquartile range, which yielded a threshold of MI = 8.93 x 10^-5^. For the calculation of the MI on GC peaks for a given direction, we used the simultaneous phase and amplitude of time windows spanning 1 s before and after the 10 highest GC peaks and computed the MI for this subset of data as described above. As a control we used the same analysis for each animal/period and computed the average of 20 comodulogram maps, each produced by windows centered at 10 random data points selected from a pool containing only data points of each REM episode with GC absolute difference between directions lower than the 5^th^ percentile.

1 Brankack, J. *et al.* Distinct features of fast oscillations in phasic and tonic rapid eye movement sleep. *Journal of sleep research* **21**, 630-633, doi:10.1111/j.1365-2869.2012.01037.x (2012).

2 Tort, A. B. L., Komorowski, R., Eichenbaum, H. & Kopell, N. in *Journal of neurophysiology* Vol. 104 1195-1210 (2010).

Supplementary Figures

**
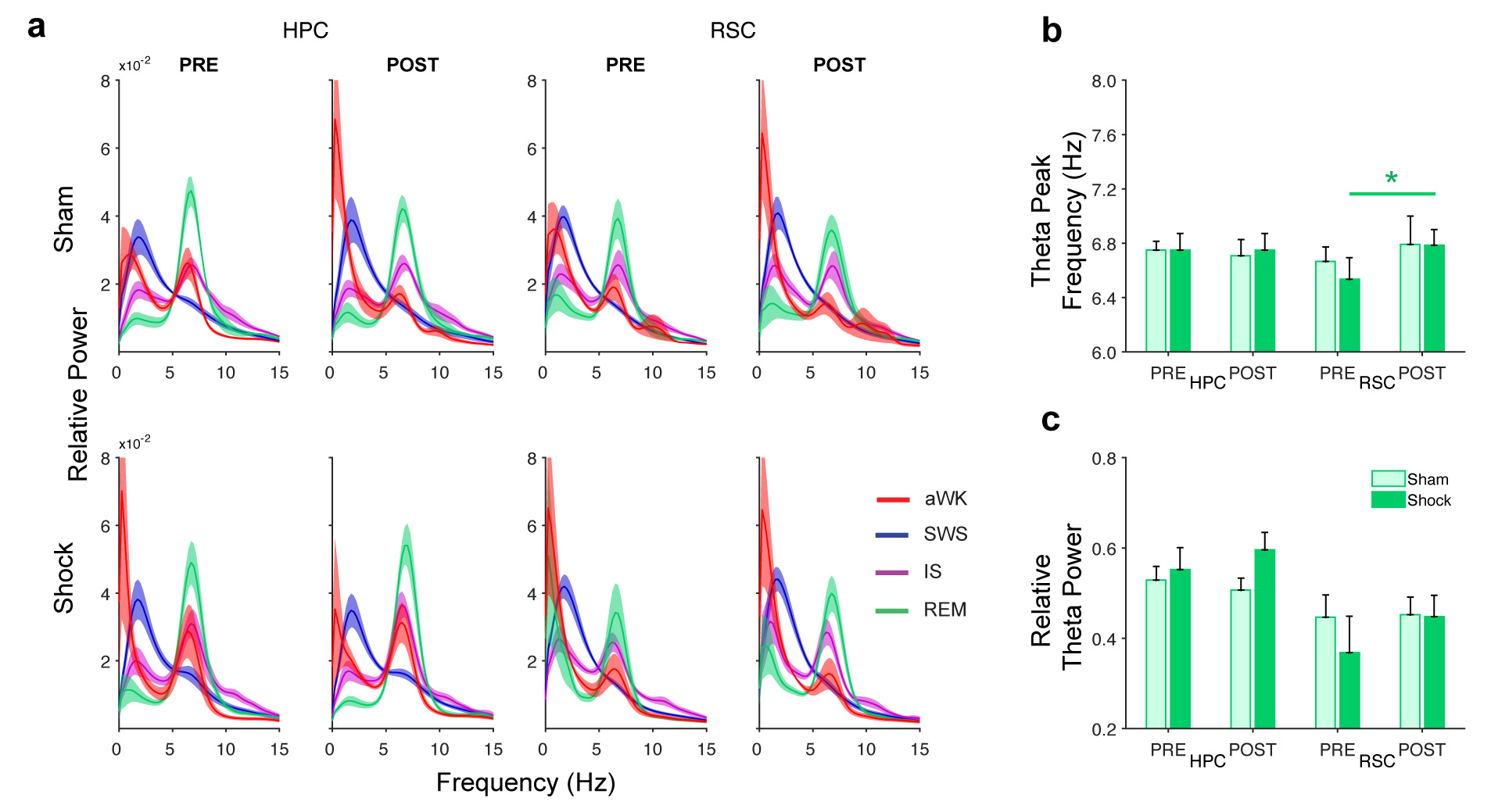
**

**Fig. S1 | LFP frequency and amplitude during different behavioral states, and especially during REM sleep. a** The relative power of low-frequency oscillations spanning from 0 to 15 Hz within selected channels, across all behavioral states. **b,c** Theta peak frequency (**b**) and Relative theta power (**c**) of HPC and RSC selected channels during REM sleep of Sham and Shock groups during pre and post. Theta peak frequency increased significantly in the RSC channel from pre to post CFC only in the shock group (RSC-shock, p = 0.04; RSC-sham, p = 0.43). No significant difference was found in the HPC channel (pre vs post: HPC-sham, p = 1.0; HPC-shock, p = 1.0. Sham, n = 6, Shock, n = 7; Wilcoxon signed rank test). No significant difference was found in relative theta power between pre and post (pre vs post: HPC-sham, p = 0.32; HPC-shock, p = 0.21; RSC-sham, p = 0.84; RSC-shock, p = 0.46. Sham, *n = 6*, Shock, *n = 7*; Wilcoxon signed rank test). No significant difference was found in unpaired (Wilcoxon rank-sum test) comparisons between groups as well (Sham, *n = 6*, Shock, *n = 7*; Sham vs Shock: HPC-pre, p = 0.95; HPC-post, p = 0.11; RSC-pre, p = 0.63; RSC-post, p = 0.95). Graphs show mean ± SEM (lines/bars ± shades/lines).

**
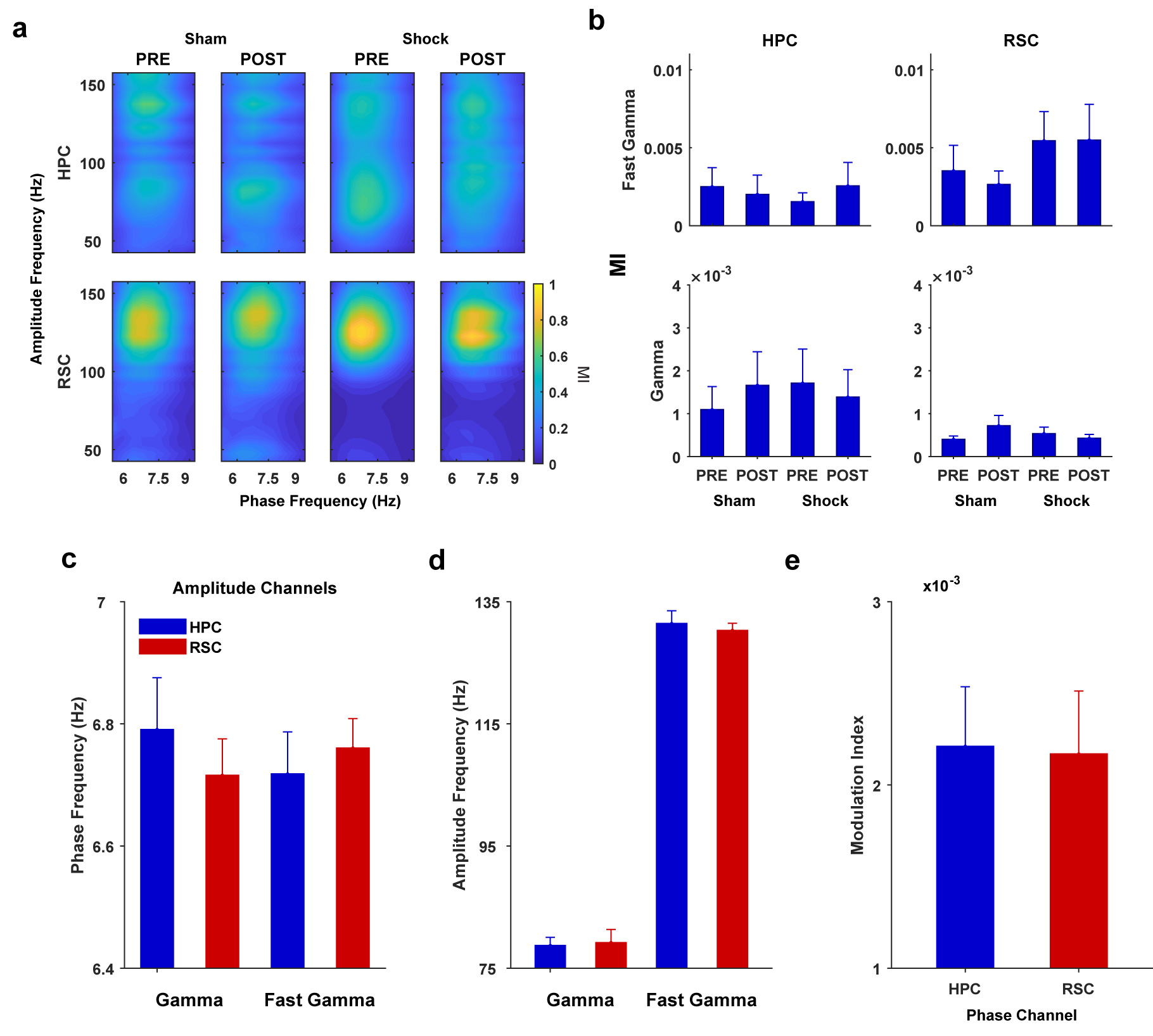
**

**Fig. S2 | Phase-amplitude coupling during total REM sleep within and between the HPC and the RSC.** **a** Comodulogram maps for HPC and RSC channels normalized by the maximum modulation index (MI) value and averaged across groups/periods during total REM sleep. HPC as the phase channel. Phase frequencies span the theta range (5 – 10 Hz), whereas amplitude frequencies encompass high-frequency oscillations ranging from 40 to 160 Hz (gamma: 40 – 100 Hz and fast gamma: 100 – 160 Hz). **b** High-frequency oscillations peak MI values in the HPC and RSC channels modulated by the HPC theta phase for each group/period during total REM sleep. In average, comodulation patterns are significant but invariant across groups and periods (limit for significance = 8.93 x 10^-5^; see Methods; n = 6 (sham) or 7 (shock) for bars; one set of tests for each combination of high-frequency oscillation with amplitude channel, *i.e.*, each one of the 4 plots in **b**). Gamma-HPC: pre vs post; sham, p = 0.82; shock, p = 0.22. sham vs shock; pre, p = 1.0; post, p = 0.76. Gamma-RSC: pre vs post; sham, p = 0.19; shock, p = 0.69. sham vs shock; pre, p = 0.79; post, p = 0.66. Fast Gamma-HPC: pre vs post; sham, p = 0.31; shock, p = 0.69. sham vs shock; pre, p = 0.44; post, p = 0.53. Fast Gamma-RSC: pre vs post; sham, p = 0.82; shock, p = 0.82. sham vs shock; pre, p = 0.53; post, p = 0.35. sham vs shock, Wilcoxon rank sum test; pre vs post, Wilcoxon signed rank test). **c**,**d** Phase frequency (**c**) and amplitude frequency (**d**) of the peak MI within each comodulogram map averaged across all conditions. HPC and RSC labels represent channels in which the amplitude of high-frequency oscillations (gamma and fast gamma) is modulated. Gamma and fast gamma oscillations within the HPC and the RSC were modulated by similar theta frequency (*n = 52* for each bar; *two-way* ANOVA for regions and fast oscillation band. Mean frequencies were gamma: HPC, 6.79 ± 0.09 Hz; RSC, 6.72 ± 0.06 Hz, and fast gamma: HPC, 6.72 ± 0.07 Hz; RSC, 6.76 ± 0.05 Hz; **c**). Likewise, the gamma and fast gamma band frequencies modulated by theta oscillations within the HPC was also similar to their respective counterparts (gamma and fast gamma, respectively) within the RSC (*n = 52* for each bar; paired *t* test. Mean frequencies were gamma: HPC, 78.7 ± 1.3 Hz; RSC, 79.2 ± 2.1 Hz, and fast gamma: HPC, 131.4 ± 2.1 Hz; RSC, 130.3 ± 1.1 Hz; **d**). **e** The peak modulation index (MI) averaged across all possible conditions, comparing the HPC vs RSC as the phase channel show that comodulation is comparable despite the phase channel (only animals/periods with significant MI values were considered*, n = 93*, *t_(92)_* = 0.6, *p =* 0.55; paired *t* test). Graphs show mean ± SEM (bars ± lines).


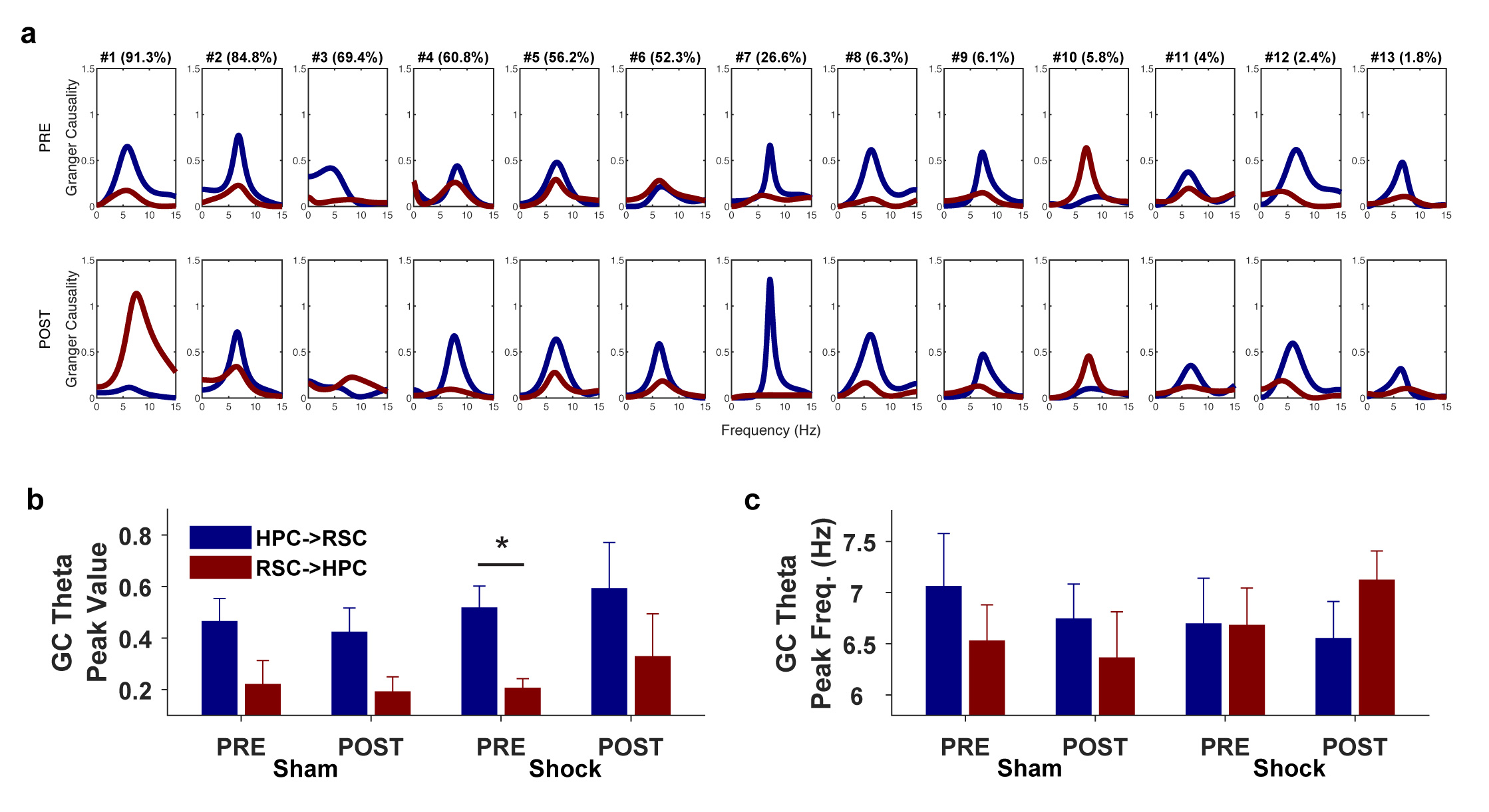


**Fig. S3 | Granger causality (GC) within theta band during REM sleep for different groups, periods, and directions. a** Granger causality value for individual animals of groups sham and shock, from 0 to 15 Hz. Animals ordered from highest to lowest freezing values (in parenthesis). **b,c** The value of GC peak within theta band during REM sleep (**b**) and its respective frequency (**c**). **b** Although GC showed a trend to be higher in the HPC🡪RSC direction in all conditions, only the shock group during pre showed a significant difference between directions (Sham, *n = 6*, Shock, *n = 7;* HPC🡪RSC vs RSC🡪HPC: pre-sham, p = 0.32; post-sham, p = 0.22; pre-shock, p = 0.03; post-shock, p = 0.30; Wilcoxon signed rank test). This suggests that the HPC leads RSC theta in most cases, but this may not be the case for all animals or all conditions as in **(a)** (see also **Fig. 2c,d**). **c** There was no significant difference in theta frequency at the peak of GC between groups, periods, or directions (Sham, *n = 6*, Shock, *n = 7*; pre vs post: HPC🡪RSC, sham, p = 0.31; HPC🡪RSC, shock, p = 0.38; RSC🡪HPC, sham, p = 0.69; RSC🡪HPC, shock, p = 0.30. HPC🡪RSC vs RSC🡪HPC: pre-sham, p = 0.69; pre-shock, p = 0.22; post-sham, p = 0.31; post-shock, p = 0.58; Wilcoxon signed rank test. sham vs shock: HPC🡪RSC-pre, p = 0.94; HPC🡪RSC-post, p = 0.84; RSC🡪HPC-pre, p = 0.94; RSC🡪HPC-post, p = 0.23; Wilcoxon rank-sum test). **p* < 0.05. Graphs show mean ± SEM (bars ± lines).


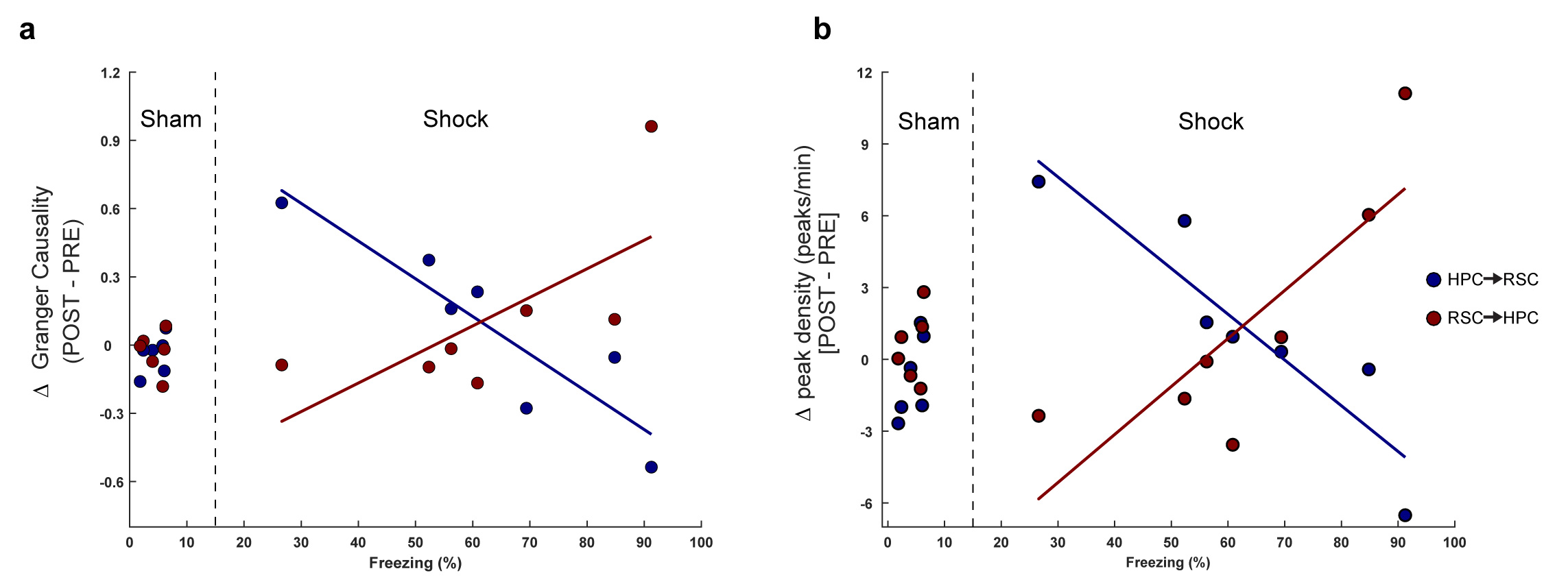


**Fig. S4 | Causality change from pre to post for both directions.** **a**,**b** For each of the animals, dots show the subtraction of the Granger causality (GC) peak value within theta band during pre REM sleep from during post REM sleep (**a**), or the subtraction of the grangerogram peak density during pre REM sleep from during post REM sleep (**b**) in the HPC🡪RSC (blue) and RSC🡪HPC (red) directions. Vertical dashed lines separate data points from animals of group sham from shock. Blue (HPC🡪RSC) and red (RSC🡪HPC) lines represent least-squares linear regressions calculated using only data points from animals of group shock. **a** The correlation between freezing percentage and change in GC is significant for the HPC🡪RSC direction (*n = 7*; *R^2^* = 0.82;
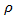
 = -0.93, *p =* 0.0067), and moderate but not significant for the RSC🡪HPC direction (*n = 7;* *R^2^* = 0.49;
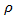
 = 0.71, *p =* 0.088; Spearman correlation). In this graph, modulation in the RSC🡪HPC direction seems lower than in the HPC🡪RSC direction, except for the animal with the highest freezing percentage. This may be explained by an actual stronger modulation in the leadership strength for the HPC🡪RSC direction. Additionally, the low dynamic variation range of GC values may play a role, especially for further decreases in the leadership strength in the RSC🡪HPC direction, which is already very low. We then plotted the grangerogram peak density change from pre to post REM sleep for both directions (**b**), as this variable shows a broader dynamic range for variation (**Table 2**). The results show that the RSC🡪HPC direction (*n = 7;* *R^2^* = 0.71;
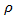
 = 0.79, *p =* 0.048) is also significantly modulated as the HPC🡪RSC direction (*n = 7;* *R^2^* = 0.68;
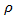
 = -1, *p =* 0.004). This suggests that the CFC protocol weakened the influence of the HPC upon RSC theta oscillation, simultaneously to the strengthening in the opposite direction in high performance animals, whereas the other animals showed an opposite trend.


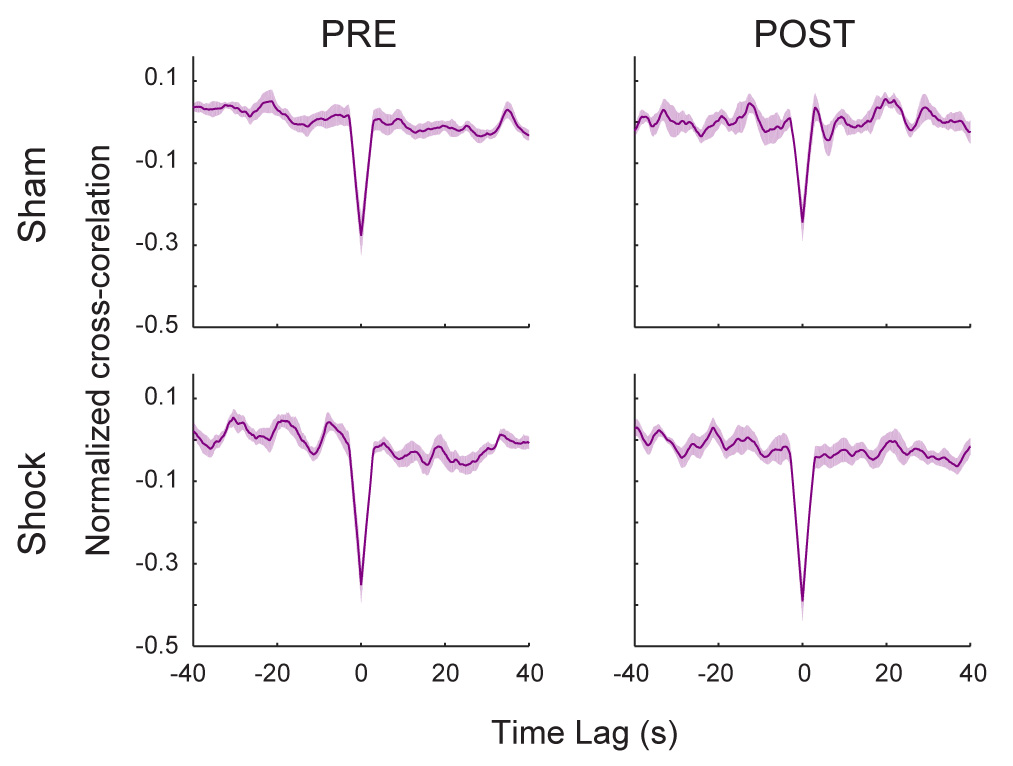


**Fig. S5 | Cross-correlation between grangerogram directions.** Average normalized cross-correlation functions between grangerogram directions across animals from sham and shock groups during pre or post. Results show a sharp negative correlation with zero lag for all conditions. No significant difference was found between periods or groups (Sham, *n = 6*, Shock, *n = 7;* pre vs post: sham, p = 0.22; shock, p = 0.47. sham vs shock: pre, p = 0.46; post, p = 0.30; **Table 2**). Graphs show mean ± SEM (lines ± shades).

**
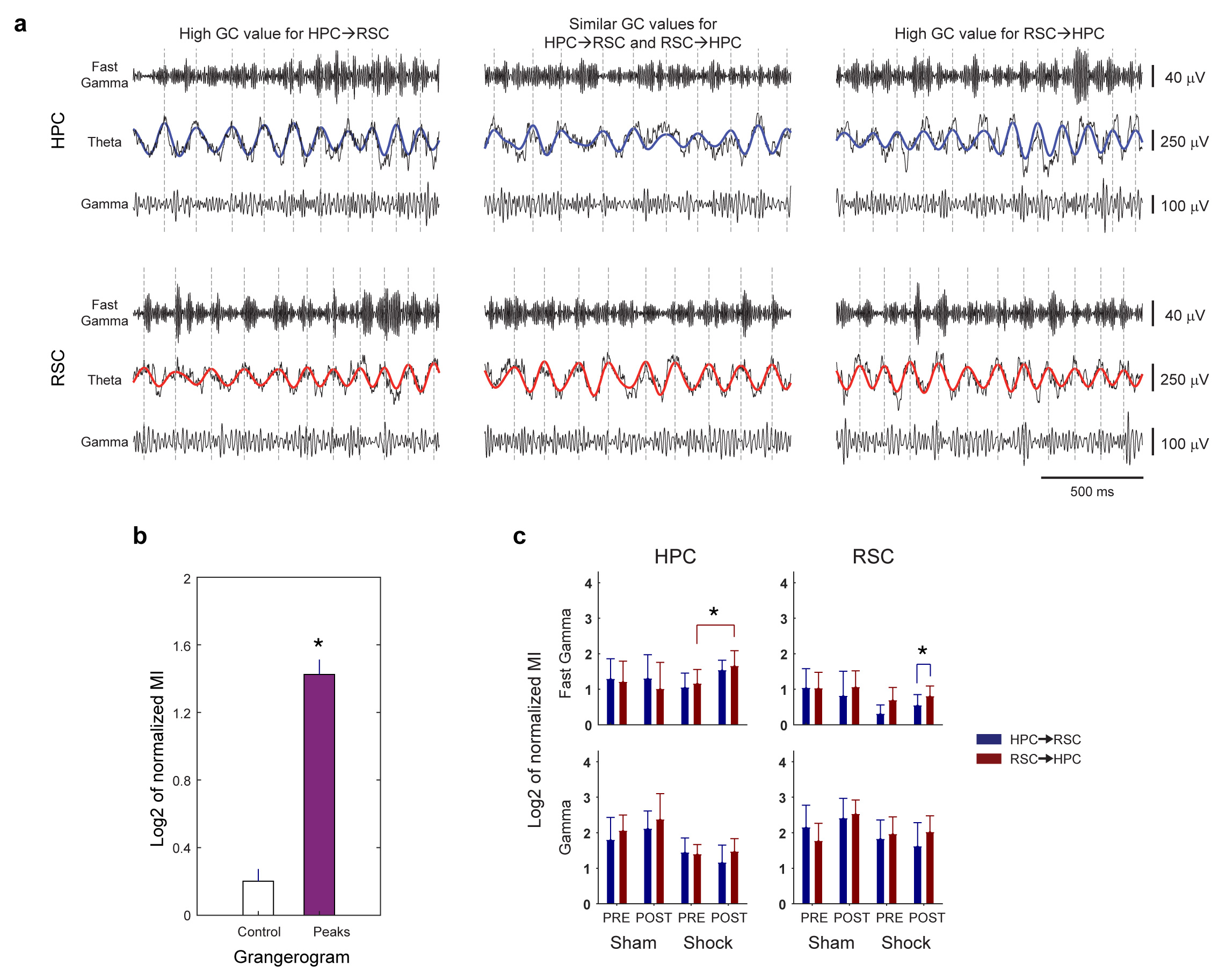
**

**Fig. S6 | Comodulation is increased in the vicinity of grangerogram peaks.** **a** 1.5 s of LFP raw traces from the HPC and RSC, and filtered in the theta, gamma, and fast gamma frequency bands in a window centered at 2 grangerogram peaks (left, HPC🡪RSC and right, RSC🡪HPC), and a data point with minimal GC difference between directions (center). Grey dashed lines, theta peaks. **b** Log2 of normalized peak MI values calculated in the vicinity of grangerogram peaks (both directions) compared with data points with minimal GC difference between directions (control; see Methods). Each data point refers to the MI of high-frequency oscillations (gamma or fast gamma) in a single channel (HPC or RSC), during one period (pre or post) from a single animal averaged across the 10 highest peaks of the grangerogram in a specific direction (HPC🡪RSC or RSC🡪HPC). Data points are normalized by the respective peak MI value of the comodulogram maps from total REM sleep during pre-CFC. This analysis revealed that comodulation is much stronger during grangerogram peaks (*n = 208*/group; *t_(207)_* = -16.9; *p =* 10^-41^; paired *t* test). **c** Stratification of comodulation patterns during GC peaks by region, period, band of high-frequency oscillation, direction, and groups. No significant difference was found between groups, periods or directions in gamma band amplitude modulation by theta phase on HPC or RSC channels [Sham, *n = 6*, Shock, *n = 7*. One set of tests for each combination of high-frequency oscillation with amplitude channel, *i.e.*, each one of the 4 plots in **c.** (HPC: pre vs post: HPC🡪RSC, sham, p = 0.31; HPC🡪RSC, shock, p = 0.47; RSC🡪HPC, sham, p = 0.85; RSC🡪HPC, shock, p = 0.58. HPC🡪RSC vs RSC🡪HPC: pre-sham, p = 0.57; pre-shock, p = 1.0; post-sham, p = 0.69; post-shock, p = 0.47; Wilcoxon signed rank test. sham vs shock: HPC🡪RSC-pre, p = 0.95; HPC🡪RSC-post, p = 0.13; RSC🡪HPC-pre, p = 0.29; RSC🡪HPC-post, p = 0.44; Wilcoxon rank-sum test.), (RSC: pre vs post: HPC🡪RSC, sham, p = 0.31; HPC🡪RSC, shock, p = 0.69; RSC🡪HPC, sham, p = 0.16; RSC🡪HPC, shock, p = 0.57. HPC🡪RSC vs RSC🡪HPC: pre-sham, p = 0.55; pre-shock, p = 0.69; post-sham, p = 1.0; post-shock, p = 0.37; Wilcoxon signed rank test. sham vs shock: HPC🡪RSC-pre, p = 0.94; HPC🡪RSC-post, p = 0.53; RSC🡪HPC-pre, p = 0.83; RSC🡪HPC-post, p = 0.53; Wilcoxon rank-sum test)]. Fast gamma modulation by theta rhythm in the HPC channel during RSC🡪HPC peaks significantly increased from pre to post only for the shock group (p = 0.01). Consistent with this change, the modulation in the same RSC🡪HPC peaks in the RSC channel was significantly stronger than peaks in the opposite direction during post also only for the shock group (p = 0.04). No other significant difference was found between groups, periods, or directions in fast gamma band amplitude modulation by theta phase on HPC or RSC channels [Sham, *n = 6*, Shock, *n = 7*. (HPC: pre vs post: HPC🡪RSC, sham, p = 0.84; HPC🡪RSC, shock, p = 0.30; RSC🡪HPC, sham, p = 0.56. HPC🡪RSC vs RSC🡪HPC: pre-sham, p = 0.84; pre-shock, p = 0.94; post-sham, p = 1.0; post-shock, p = 1.0; Wilcoxon signed rank test. sham vs shock: HPC🡪RSC-pre, p = 0.83; HPC🡪RSC-post, p = 0.45; RSC🡪HPC-pre, p = 0.94; RSC🡪HPC-post, p = 0.53; Wilcoxon rank-sum test.), (RSC: pre vs post: HPC🡪RSC, sham, p = 0.85; HPC🡪RSC, shock, p = 0.69; RSC🡪HPC, sham, p = 0.85; RSC🡪HPC, shock, p = 0.94. HPC🡪RSC vs RSC🡪HPC: pre-sham, p = 1.0; pre-shock, p = 0.82; post-sham, p = 0.69; Wilcoxon signed rank test. sham vs shock: HPC🡪RSC-pre, p = 0.63; HPC🡪RSC-post, p = 0.95; RSC🡪HPC-pre, p = 0.53; RSC🡪HPC-post, p = 1.0 Wilcoxon rank-sum test)]. Graphs show mean ± SEM (bars ± lines).


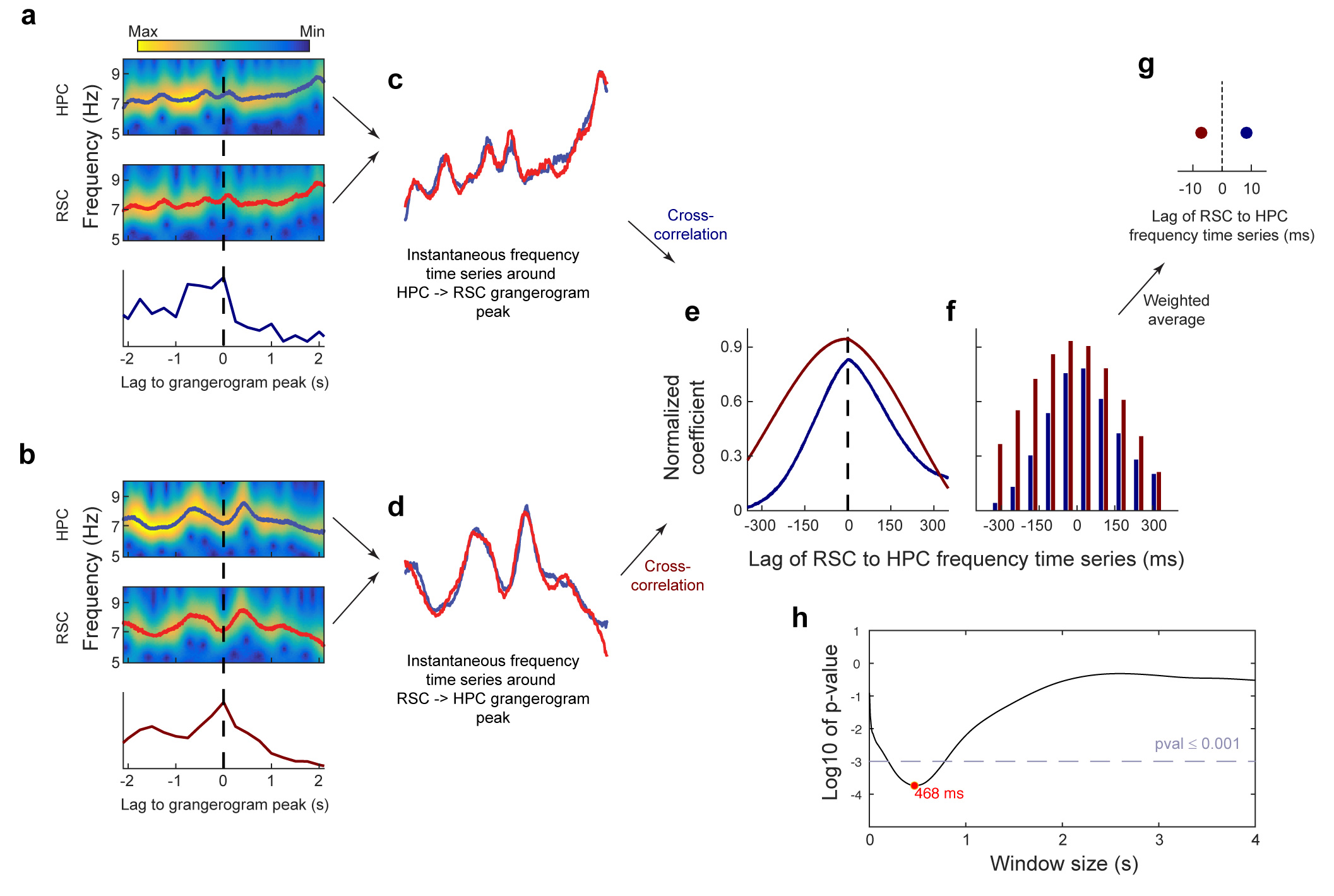


**Fig. S7 | Calculation of the lag of theta acceleration between HPC and RSC channels in the vicinity of grangerogram peaks.** **a**,**b** Wavelet spectral decomposition of HPC and RSC time series (top and middle, respectively) in the vicinity of HPC🡪RSC (**a**, bottom) and RSC🡪HPC (**b**, bottom) exemplary grangerogram peaks (± 2 s). **c**,**d** Instantaneous theta frequency time series from both regions (HPC, light blue and RSC, light red) acquired by the detection of the peak frequency for each time point from (**a**), depicted in (**c**), and from (**b**), depicted in (**d**). **e** Cross-correlation functions plotted from -330 to +330 ms in relation to zero lag to the HPC time series, as calculated using the instantaneous theta frequency time series in the vicinity of the HPC🡪RSC (dark blue) grangerogram peak (**a** and **c**), and the RSC🡪HPC (dark red) grangerogram peak (**b** and **d**). **f** The average of cross-correlation functions across 66 ms windows (no overlap) plotted as bars to highlight the asymmetry of cross-correlation functions. **g** The weighted time lag average for each direction (HPC🡪RSC, dark blue and RSC🡪HPC, dark red), using the values of the cross-correlation functions as weights. The plot shows weighted averages laying at different time lags expressing the different and asymmetric patterns of cross-correlation functions related to grangerogram peaks in opposite directions. **h** To determine the window size which maximizes the statistical difference between weighted time lag averages from both directions, we computed the unpaired *t* test for these variables using window sizes from 2 to 4000 ms. The plot of the log10 of *p* values in relation to the window sizes shows that the window size of 468 ms (± 234 ms in relation to zero lag) elicits the lower *p* value.


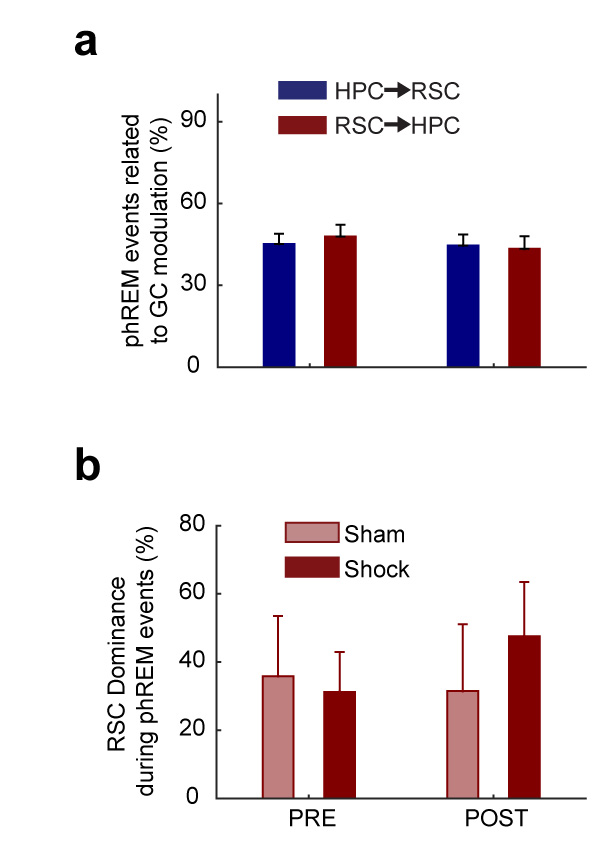


**Fig. S8 | Characteristics of GC modulation during phREM events. a** Percentage of phREM events with positive or negative GC modulation respective to each direction showing no difference between directions or type of modulation (positive vs negative; see Methods) (sham, *n = 6*, shock, *n = 7;* HPC🡪RSC vs RSC🡪HPC: positive, p = 0.60; negative, p = 0.85. positive vs negative: HPC🡪RSC, p = 0.61; RSC🡪HPC, p = 0.27). **b** The percentage of phREM events with RSC dominance (RSC🡪HPC > HPC🡪RSC direction) also showed no difference when averaged within groups and periods (Sham, *n = 6*, Shock, *n = 7*; pre x post: sham, p = 0.42; shock, p = 0.79. sham vs shock: pre, p = 0.71; post, p = 0.15). Graphs show mean ± SEM (bars ± lines).


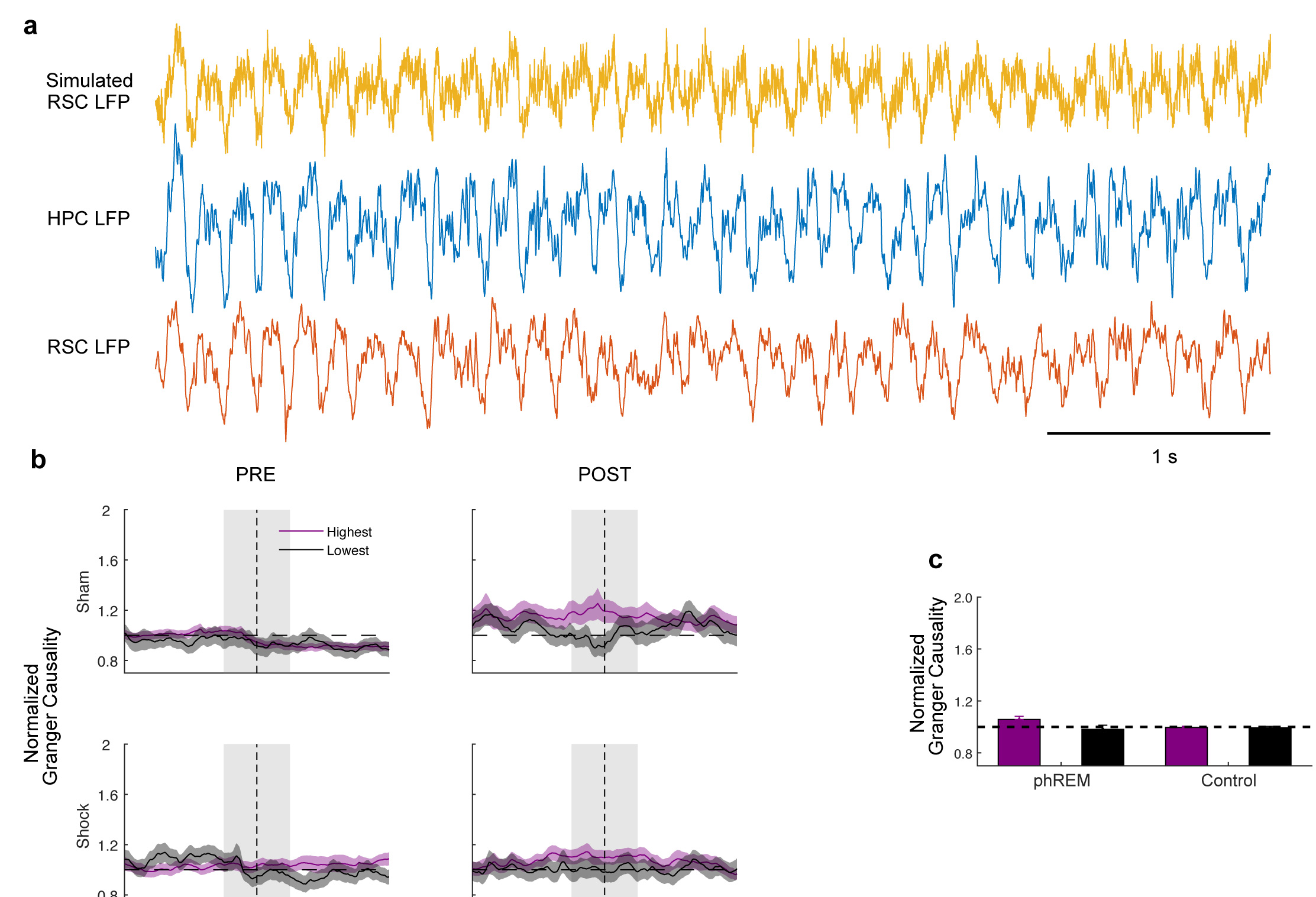


**Fig. S9 | Checking whether transient theta acceleration induces spurious GC increase.** **a** Representative traces of original and simulated LFP during a REM sleep episode. Top, simulated RSC LFP (see Methods). Middle and bottom, original HPC and RSC simultaneously recorded LFP, respectively. **b** The grangerogram traces centered at phasic REM events, as depicted in **Fig. 4d**, but using the original HPC and simulated RSC traces as input for GC calculation. **c** Normalized GC averaged across one phREM unit as in **Fig. 4e**, but using the simulated RSC LFP, showed no difference when compared to controls (random data points). Black dashed line, baseline. Note that, contrary to the calculations using the original data depicted in **Fig. 4d**, transient theta acceleration during phasic REM events do not modulate GC calculated using a simulated RSC trace. (*n = 396*/group. Highest: phREM x control, *t_(790)_* = -1.7, *p =* 0.08; Lowest: phREM x control, *t_(790)_* = -0.9, *p =* 0.37; unpaired *t* test). Graphs show mean ± SEM (lines/bars ± shades/lines).


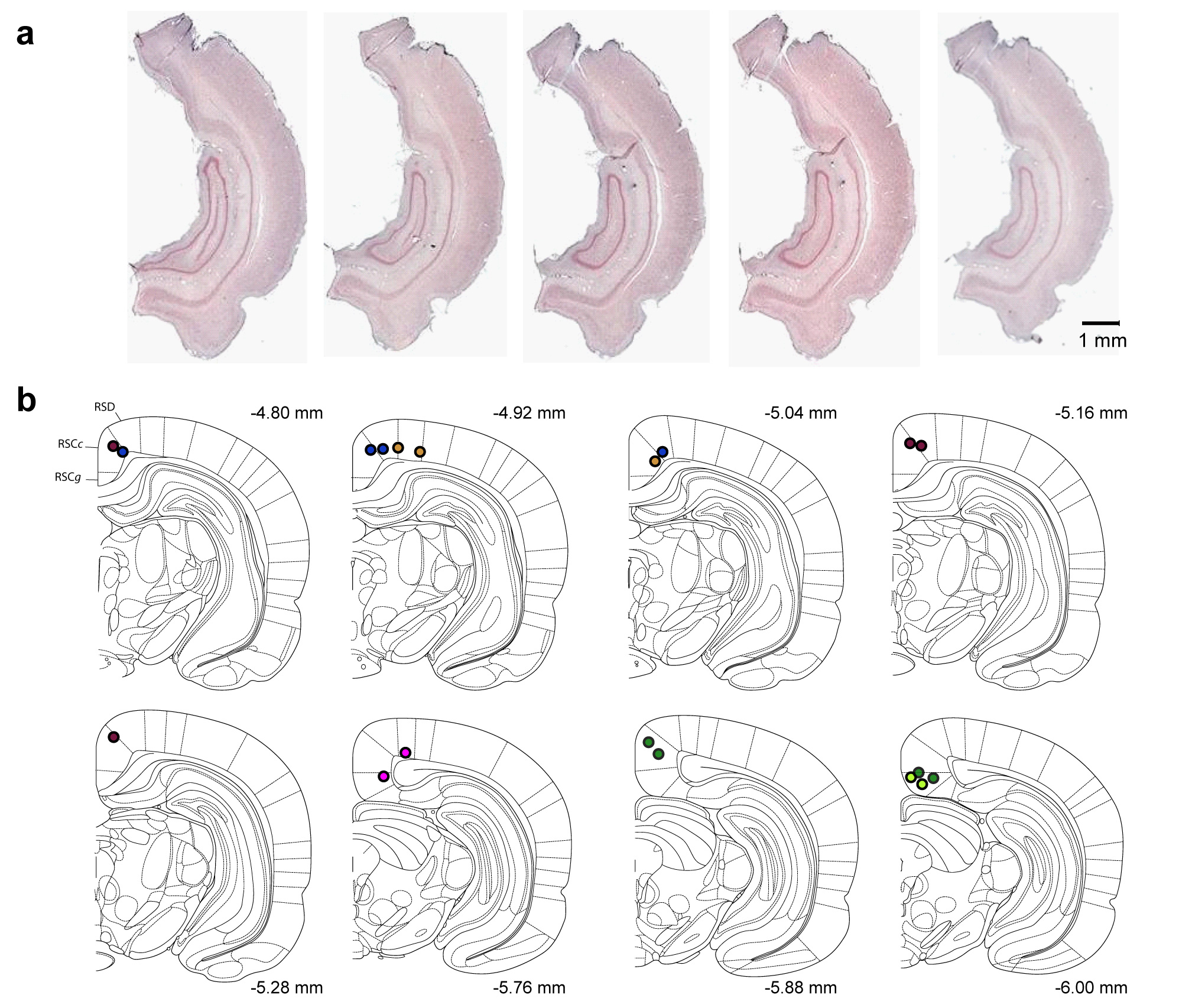


**Fig. S10 | Histological analysis of electrode locations.** **a** One representative example of the sections used to reconstruct electrode positions. **b** Localization of the electrodes used in our study.


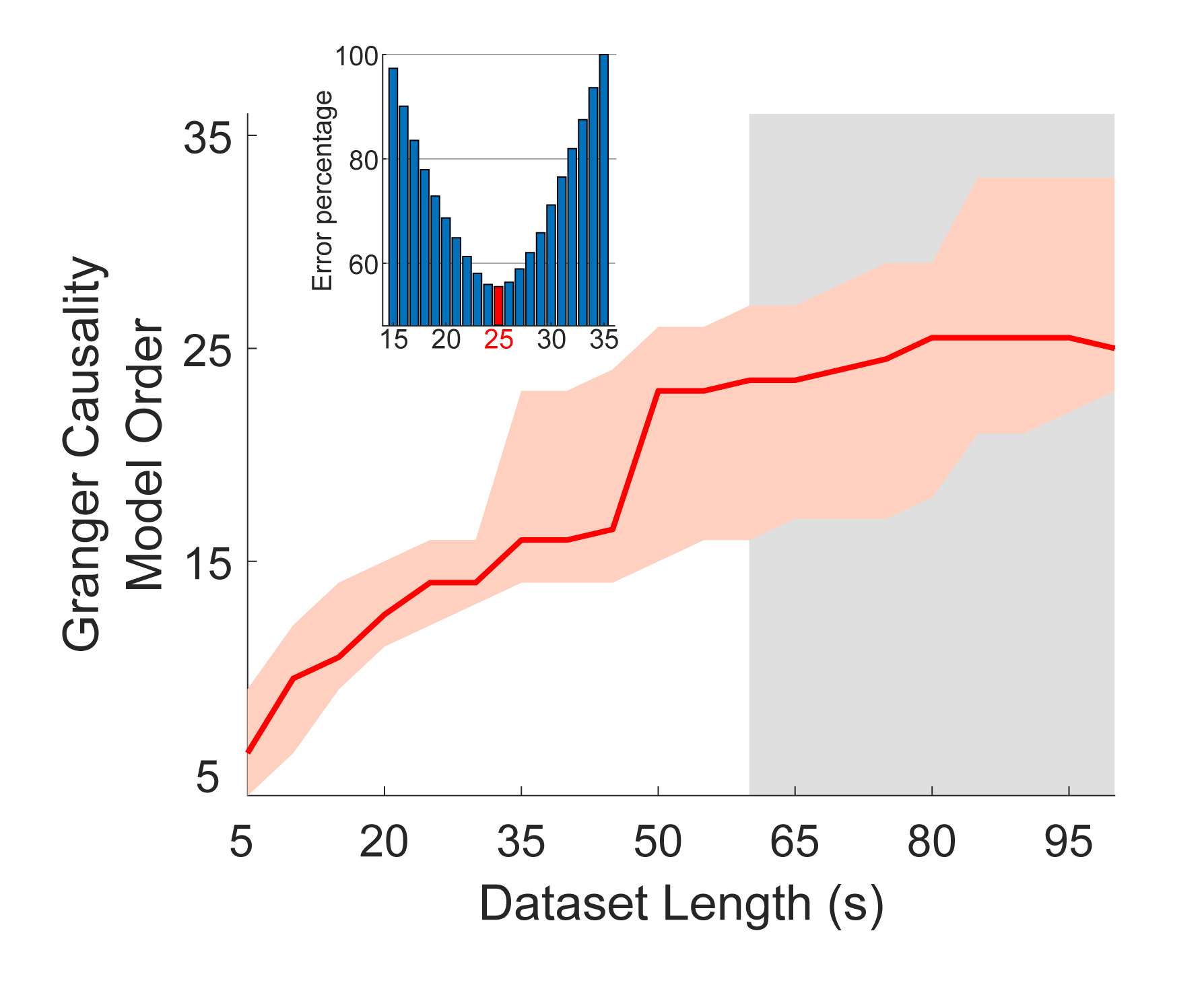


**Fig. S11 | Definition of the model order for GC calculations.** Plot showing the relation between REM sleep dataset length (s) and the model order computed using the default parameters of the MVGC toolbox (see Methods). Red line represents the median and the red shaded area are limited up and down by the 3º and 1º quartiles, respectively. Grey shaded area highlights dataset lengths ≥ 60s, in which the median of model orders seem to reach a plateau around the value of 25. The inset shows the error percentage between the model order calculated for all animals/periods on data set lengths spanning the grey shaded area (60-100 s) and specific model order values ranging from 15 to 35. Error percentage values were normalized by the maximum error (model order = 35; mean error = 5.74). Note the u-shaped curve with the through on a model order = 25, indicating that this model order seems ideal for the estimation of GC across different animals/periods.
